# Metabolic and gut microbiota effects of ketogenic diet and exogenous ketone salts in a rat model of metabolic syndrome

**DOI:** 10.1007/s00394-026-03967-z

**Published:** 2026-04-28

**Authors:** Alexandre Pereira-Rodrigues, Alexandre Gonçalves, Inês N. Alves, Cláudia Sousa Mendes, Carolina Silva, Joana Campos, Benedita Sampaio-Maia, Inês Falcão-Pires, Ricardo Araujo

**Affiliations:** 1 INEB-Institute of Biomedical Engineering, University of Porto, Rua Alfredo Allen 208, 4200-135 Porto, Portugal; 2 i3S-Institute for Research & Innovation in Health, University of Porto, Rua Alfredo Allen 208, 4200-135 Porto, Portugal; 3 Department of Surgery and Physiology, Faculty of Medicine, University of Porto, Alameda Prof. Hernâni Monteiro, 4200-319 Porto, Portugal; 4 Department of Surgery and Physiology, Faculty of Medicine, RISE-Health, University of Porto, Alameda Prof. Hernâni Monteiro, 4200-319 Porto, Portugal; 5 Faculdade de Medicina Dentária, Universidade do Porto, R. Dr. Manuel Pereira da Silva, 4200-393 Porto, Portugal

**Keywords:** Gut microbiome, Ketogenic diet, Metabolic syndrome, Nutrition, Obesity, Rat model

## Abstract

**Purpose:**

Metabolic syndrome (MetS) represents a significant public health challenge, with emerging evidence pointing to gut microbiota as a key player in its development and progression. This study explored the comparative effects of ketogenic diet (KD) and ketone salts supplementation (KS) on metabolic dysfunction and gut microbiota composition in ZSF1 obese rats, an established MetS rat model.

**Methods:**

ZSF1 obese and lean rats were allocated to a control diet, a ketogenic diet or ketone salts supplementation. Metabolic assessments and 16 S rRNA V4 region sequencing (Illumina Miseq) were performed to evaluate glucose metabolism and gut microbiota composition.

**Results:**

Our validation confirmed the MetS phenotype in this model, including increased body weight and adiposity, which were further amplified in rats receiving the ad libitum KD. Although both KD and KS effectively reduced fasting glucose levels, the KD, contrary to its typical weight-reducing effect in humans, significantly increased body weight and mesenteric fat, whereas KS did not alter adiposity relative to the control diet. Both interventions profoundly impacted the gut microbiota profile; KD reduced microbial richness and shifted community composition (including a lower Bacillota/Bacteroidota ratio and higher *Akkermansia* levels). In contrast, the intervention with KS resulted in a gut microbiota profile resembling the control rats fed with the control diet.

**Conclusion:**

These results highlight the distinct effects of different ketogenic interventions (KD and KS) on host metabolism and gut microbiome, suggesting that while both can influence glucose control, KS may offer a more favorable metabolic and microbiota profile for managing MetS, without the strict dietary adherence required by KD.

**Supplementary Information:**

The online version contains supplementary material available at 10.1007/s00394-026-03967-z.

## Introduction

The silent epidemic of noncommunicable diseases, accounting for 74% of global deaths annually, represents an urgent threat to public health [1]. Within this context, metabolic syndrome (MetS), a complex constellation of interconnected risk factors, including hypertension, obesity, diabetes, and dyslipidemia, stands out as a particularly intricate challenge. Recent evidence highlights the gut microbiota as an important contributor to metabolic regulation, with characteristic shifts in microbial diversity and specific taxa reported in obesity and type 2 diabetes [1, 2]. Several genera, such as *Blautia*, *Coprococcus*, and *Ruminococcus*, have been associated with this obesity-related metabolic dysfunction, while beneficial taxa, including *Alistipes* (Bacteroidota), *Faecalibacterium*, *Oscillibacter* (Bacillota), and *Akkermansia* (Verrucomicrobiota) were tendentially reduced in individuals with obesity or MetS [3–5].

Diet plays a major role in the development and stability of the microbiome, and its modulation presents an opportunity to improve the composition of gut microbiota and its health benefits. Dysbiosis has been linked to MetS, and nutrition underlies the gut microbiome’s homeostasis [6, 7]. Thus, understanding how nutrition and changes in the proportion of macro- and micronutrients impact gut microbiota is critical for determining its potential efficacy in MetS. As previously discussed by our group, the beneficial effects of the ketogenic diet (KD) may be partly mediated by changes in gut microbiota composition, in particular with the growth of beneficial bacteria, such as *Akkermansia*, *Muciniphila*, and *Lactobacillus*, and the reduction in pro-inflammatory microbes, *Desulfovibrio* and *Turicibacter* [8]. Conversely, it has been demonstrated that while a KD induces hyperketonemia (elevated circulating beta-hydroxybutyrate), it also disrupts the gut microbial community by decreasing overall bacterial diversity and richness, indicating potential adverse effects on the gut ecosystem [9].

Despite concerns regarding gut microbial diversity, KD remains a promising dietary intervention for addressing metabolic dysregulation associated with MetS. Characterized by its high-fat and very low-carbohydrate composition, KD promotes a metabolic shift towards fatty acids oxidation and ketones production, which can influence glucose homeostasis and energy balance [8, 10–12]. KD has been associated with weight reduction and improved glycemic control in humans. Effects are attributed to increased fat oxidation, reduced appetite, and preservation of lean mass [13]. Although several studies report a reduction in circulating inflammatory markers in KD, these effects may depend on diet composition, duration, and baseline metabolic status [12, 14, 15]. Importantly, some KD-associated immune changes appear to occur through shifts in gut microbiota, including alterations in Th17 cell populations, but these mechanisms remain under investigation and were not directly assessed in the present study [16].

However, the highly restrictive nature of KD limits patient compliance. Furthermore, KD could initially raise blood cholesterol and free fatty acids, leading to increased body acidosis, primarily due to the accumulation of β-hydroxybutyrate (BHB) and acetoacetate [17]. Exogenous ketone supplementation, typically in the form of ketone esters (KE) or ketone salts (KS), offers a potential alternative for achieving ketosis without strict carbohydrate restriction [18]. KS formulations have been shown to elevate circulating BHB and acetate concentrations in healthy adults [19]. Beyond their role as energy substrates, ketones have been reported to influence cellular signalling pathways, including those related to metabolic regulation and immune function [20], though these mechanisms were not examined in the present study.

The intricate pathogenesis of MetS, involving a complex interplay of genetic and environmental factors, requires robust animal models for comprehending its pathophysiologic mechanisms. The ZSF1 obese rat, with its inherent predisposition to metabolic dysregulation, mirrors key features of human MetS, including obesity, hyperglycemia, dyslipidemia, and reduced exercise tolerance, allowing for the exploration of how gut microbiota alterations exacerbate these conditions [21]. This model also enables analysis of how dietary interventions influence both metabolic dysfunction and gut microbiota composition. Given the known association between gut microbiota alterations and impaired metabolic regulation, we hypothesized that KD and KS would differentially modify the gut microbiota and influence metabolic outcomes in ZSF1 rats.

Thus, we aim to determine whether KD or KS could modulate the gut microbiota and metabolic profile in ZSF1 rats and whether these interventions differ in their capacity to counteract MetS-associated dysbiosis.

## Materials and methods

### Animal model of MetS

Animal procedures were reviewed and approved by the Faculty of Medicine of the University of Porto (FMUP) Animal Welfare and Ethics Review Body [Órgão Responsável pelo Bem-Estar dos Animais (ORBEA-FMUP)] and the Portuguese competent authority [Direção Geral de Alimentação e Veterinária (DGAV), reference number 8161/23-S] and performed in accordance with EU Directive 2010/63/EU and Decreto-lei 113/2013 national legislation. The research was conducted at the FMUP animal facility. All procedures were carried out by properly trained and licensed researchers.

To assess the impact of nutritional ketosis on gut microbiota and metabolic function, we used 9-week-old ZSF1 male obese rats (MetS, *n* = 30), which may also develop diabetes, hypertension, and dyslipidemia, and compared them to ZSF1 male lean rats (CT = 30), which are only hypertensive and serve as a control. The animals were obtained from Charles River (USA) and housed in groups of two animals per cage, maintained on a 12-hour light-dark cycle, with full access to the recommended diet (LabDiet^®^ 5008, International Product Supplies Ltd) and water *ad libitum*. The individual ventilated chambers were maintained under controlled environment temperature (22 °C), relative humidity (30–70%), and air exchange rate (40–50 air changes per hour). At 16 weeks, animals were randomly allocated to one of three diets: standard diet (LabDiet^®^ 5008, SD), ketogenic diet (Bio-Serv F7904, KD), or standard diet supplemented with ketone salts (KS) delivered through drinking water (300 mg/100 g body weight, BW). Randomization was performed by the designated animal facility caretaker, who was not otherwise involved in the study planning or data acquisition and, therefore, impartial to group assignment. Water intake was measured regularly (1–2-week intervals) at the cage level, normalized to the number of rats per cage, and used to adjust the KS concentration to maintain the intended dose. The estimated ingested BHB dose (mg/day per animal) was calculated from water consumption and recorded body weight. These estimates and water intake profiles are provided in the supplementary material. Weight gain and energy intake were recorded every week. Metabolic assessments of the insulin resistance test (IR), oral glucose tolerance test (OGTT), and oral ketone tolerance test (OKTT) were performed by technicians blinded to intervention group, following an 8-hour fast at 14–15 weeks of age (before group separation) and at 21–22 weeks (before terminal procedures). For the OGTT, animals received an oral gavage of a 30% D-Glucose solution at 0.33mL/100 g of body weight. For the IR test, an intraperitoneal dose of 0.75 U/mL insulin solution at 0.1 ml/100 g body weight was administered. Lastly, for the OKTT, animals received an oral gavage of 3 g/kg of KS. Glucose and β-hydroxybutyrate levels were recorded at 0, 15, 30, 60, 90, and 120 min after oral or IP administration (FreeStyle Precision Neo). At 22 weeks old, we performed tolerance effort testing with maximum oxygen consumption (VO_2_max) in a closed chamber treadmill at 15° inclination coupled to a gas analyzer alongside with respiratory quotient (RQ) measurements. Daily energy intake was calculated from weekly measurements of individual food intake, together with the manufacturer-provided caloric density for each diet. Intake was averaged over each week and normalized to body weight. Rats aged 23–30 weeks were anaesthetised in ventilated chambers and euthanised while under deep anaesthesia using 8% sevoflurane delivered via a nose cone. The organs were excised and weighed, and the right tibia was also removed and measured for normalization of the organs. The samples were collected, rinsed with PBS, frozen in liquid nitrogen, and stored at − 80 °C for molecular studies or fixed in 10% buffered formalin for histological procedures. For the hemodynamic evaluation, venous blood samples collected from the RV in EDTA-containing tubes were centrifuged at 5,000 rpm for 15 min at 4 °C, and plasma was collected and frozen at − 80 °C until analysis. Plasma levels of leptin and FGF-21 were measured by an ELISA kit (Abcam and R&D systems, respectively), according to the manufacturer’s instructions. Absorbance was recorded at 450 nm using an ELISA plate reader, and linear regression was plotted and used to calculate the leptin and FGF-21 concentration in the plasma samples.

Outcome assessors for metabolic measurements, ELISAs, histology, and microbiome sequencing were blinded to group allocation throughout data collection and initial data processing. Animal labels used coded IDs, and unblinding occurred only after all primary analyses were completed.

### DNA extraction from fecal samples

Fecal samples were removed from the large intestines directly to a sterile microtube and immediately stored at -80 °C until analysis. Stool’s genomic DNA (gDNA) extraction was performed using the QIAamp Fast DNA Stool Mini Kit (QIAGEN, Germany) following the protocol with some optimization for rat stool samples [22, 23]. Firstly, the samples were weighed (100 mg) in a 2 mL microcentrifuge tube and vortexed (beadbeater) continuously for 1 min with 1.5 mL of InhibitEx Buffer. Then the samples were suspended in heat at 95 °C for cells that are difficult to lyse, such as Gram-positive bacteria. Later, the stool sample was centrifuged, and the supernatant was pipetted into a new microcentrifuge tube containing proteinase K and Buffer AL and subsequently incubated at 70 °C for 20 min. Lastly, the gDNA was cleaned with ethanol, Buffer AW1, and Buffer AW2, and then eluted in Buffer ATE (50 µL) using a QIAamp spin column, which eluted the DNA into a microcentrifuge tube. DNA quality was assessed using the NanoDrop 2000 UV spectrophotometer (Thermo Scientific, Waltham, MA, USA). To obtain superior quality samples, a minimum concentration of 10 ng/mL and a purity value greater than 1.8 in the spectrophotometer reading at a ratio of 260/280nm were required (Nanodrop Technologies, Wilmington, DE).

### 16 S rRNA amplicon sequencing

PCR amplification was accomplished using the 16 S rRNA gene universal primers targeting this bacterial gene [24]. The current primers have been modified from the original 515 F–806R primer pair in the following ways [25]. Barcodes are now on the forward primer 515 F [25]. This enables the usage of various reverse primer constructs to obtain longer amplicons. Degeneracy was added to both the forward and reverse primers to remove known biases against Crenarchaeota/Thaumarchaeota (515 F, also called 515 F-Y) [25] and the marine and freshwater Alphaproteobacterial clade SAR11 (806R) [26]. Illumina Miseq sequencing was performed for region V4 of the 16 S rRNA gene with sequencing primers as follows. Read 1 sequencing primer (forward) – TATGGTAATTGTGTGYCAGCMGCCGCGGTAA, and read 2 sequencing primer (reverse) – AGTCAGCCAGCCGGACTACNVGGGTWTCTAAT [24–26].

### 16 S rRNA gene sequence analysis

Targeted microbiota sequences were analyzed by the DADA2 pipeline [27] using filters to refine and enrich data. The raw data was analyzed in software R using DADA2 to import the raw sequences, remove the connectors and primers, and prune the sequence (retain the first 240 bp of all forward sequences and the first 200 bp of reverse sequences). The sequence information and the original abundance file of amplified subsequence variants (ASVs) were obtained after filtering, denoising, de-chimerism, and merging. After sequencing quality control and DNA-extraction filtering, the numbers of fecal samples included in the microbiome analysis were CT_SD = 10, CT_KD = 9, CT_KS = 10, MetS_SD = 7, MetS_KD = 5, and MetS_KS = 10. DNA extraction failure was primarily attributable to the lipid-rich consistency of feces from KD-fed rats, which reduced the yield of gDNA despite repeated optimization. The samples were rarefied to 19,020 reads to normalize sequencing depth and calculate the relative abundance. Taxonomic classification of ASVs was performed using the Silva version 138.2 database [28]. Profiles were compared according to the phenotype and dietary intervention at phylum, family, and genus levels. The diversity and statistical analyses were performed using R microeco [29]. Alpha Diversity metrics of the dataset were calculated with Observed, Chao1, Shannon, and Pielou indexes. Beta-diversity was analyzed using Bray–Curtis dissimilarity matrices calculated from non-rarefied ASV counts. A multivariable permutational ANOVA (PERMANOVA) was performed using adonis2 (vegan package) with 999 permutations. Differential taxonomic abundance analyses were performed at the phylum, family, and genus levels using ANCOM-BC v2 [30], which accounts for compositionality and sampling bias. Models included the combined phenotype–treatment factor, and multiple testing was controlled using the Benjamini–Hochberg false discovery rate (FDR). Taxa with FDR-adjusted q-values < 0.05 were considered statistically significant. Effect sizes are reported as log-fold changes (LFC) with directionality indicating enrichment or depletion relative to the reference group.

All 16 S rRNA amplicon sequences have been deposited in NCBI BioProject PRJNA1303361 to be released to the public on March 2026.

## Histochemistry and computerized Image

Segments of small and large intestine were harvested from ZSF1 rats and fixed in formalin, dehydrated in ethanol, cleared in xylol, and impregnated in paraffin. Three-micrometer slides were dewaxed, rehydrated, and stained with Hematoxylin-Eosin (HE) to assess microvilli area and finally mounted with Entellan^®^. An optical microscope (Leitz Wetzlar – Dialux 20, Wetzlar, Germany), equipped with a photographic camera (Olympus XC30, Tokyo, Japan), was used to visualize and photograph the histological preparations. The size of 16 microvilli per animal was measured using Cell^B software (Olympus). Histological scoring and microvilli measurements were conducted independently by two observers blinded to diet and phenotype.

### Statistical analysis

Morphometric characteristics were summarized as counts and percentages for categorical variables and mean and standard deviation (SD) for continuous variables. Two-way ANOVA (factors: phenotype × diet) with Tukey’s post hoc test was used for normally distributed outcomes. Non-parametric variables were analyzed using Kruskal–Wallis followed by Dunn’s post hoc correction. Statistical significance was defined as *p* < 0.05. For microbiome analyses, significance refers to FDR-adjusted q-values unless otherwise specified. For the primary metabolic endpoint (fasting glucose), all animals (*n* = 10 per group) were included in the analysis. Effect sizes (Cohen’s d and η²) with 95% confidence intervals are reported, and post-hoc power estimates based on observed means, standard deviations and sample sizes are provided for descriptive purposes in Tables S1 and S2.

## Results

### Ketogenic diet and exogenous ketone salt supplementation improve glucose in MetS

To verify the development of MetS in ZSF1 rats, body weight, mesenteric fat, glucose metabolism, and several plasma metabolites were evaluated. Body weight and mesenteric fat were significantly higher in the MetS model compared to the control (CT group), confirming obesity (*p* < 0.001 for both, Table 1). MetS rats showed a significantly higher level of glucose and fasting glucose compared to the controls (*p* < 0.001 for both, Table 1). In parallel with hyperglycemia, the IR test was significantly higher in MetS rats compared to the control (*p* < 0.0001, Table 1). OGTT was markedly greater in MetS rats (*p* < 0.001), in parallel with high plasma glucose levels, proving the development of impaired glucose metabolism (Table 1). Thus, these results confirm the presence of diabetes in MetS rats. FGF-21 and leptin levels were also elevated in the MetS rats, consistent with severe metabolic dysregulation in obesity and diabetes (Table 1). We confirmed that MetS impacted exercise tolerance, as evidenced by lower maximal oxygen consumption (VO2max, *p* < 0.001), energy expenditure (EE, *p* < 0.001), and shorter running distances (*p* < 0.001; Table 1). To understand if both interventions impact the MetS rat models, the same morphometric parameters mentioned above were assessed. KD significantly increased body weight and mesenteric fat (*p* = 0.0013 and *p* = 0.0061, Table 1). In contrast, KS did not significantly alter body weight or mesenteric fat relative to the standard diet, indicating a metabolically neutral effect on adiposity. MetS_SD animals consumed significantly more calories compared to the MetS_KD and MetS_KS groups (*p* < 0.001, Figure S1), yet MetS_KD exhibited higher body weight and mesenteric fat (Table 1). Differences between KD and KS were paralleled by hormonal changes, with KD markedly elevating FGF-21 and leptin (*p* = 0.0015 and *p* < 0.0001), whereas KS did not statistically differ from the standard diet (Table 1). Both interventions lowered glucose (*p* < 0.0001 for both) and fasting glucose levels (*p* = 0.0043 for KD and *p* = 0.0115 for KS), while the IR test and OGTT responses remained unchanged relative to the standard diet (Table 1). Exercise capacity variables (VO_2_max, EE, and total running distance) also showed no improvement with either intervention (Table 1). RQ values were significantly higher in MetS_SD animals relative to CT (*p* = 0.006, Figure S1), consistent with increased fat oxidation. Additionally, we performed ketone tolerance tests (KTT) and ketone level measurements to assess if MetS changed ketone usage capacity and if ketogenic diet (KD) or salt (KS) interventions were providing ketones as an alternative fuel under dysmetabolic conditions. KD significantly increased circulating ketone levels and KTT responses in both phenotypes (*p* < 0.0001), confirming robust nutritional ketosis (Table 1 and S3). In contrast, KS produced only modest increases, consistent with expected pharmacokinetics of exogenous salts (Table 1 and S3). Although peak blood BHB levels were lower in KS than with the KD, this does not preclude their metabolic and modulatory effects. Driven by increased uptake, the body can quickly blunt the detectable increase in blood BHB that would be seen with exogenous salts, while nutritional ketosis provides a stable increase in these levels. Daily water intake was consistent with well-described patterns in the ZSF1 model and remained stable across the study (Figure S2). Based on measured intake and adjusted solution concentrations, MetS rats consistently ingested higher absolute amounts of BHB than the control rats (Table S4).

**Table 1:** Morphological characterization of the rat model groups. Weights were recorded at the time of sacrifice

Characteristic	Control (CT)			Metabolic Syndrome (MetS)		
	Control Diet (CT_SD)	Ketogenic Diet (CT_KD)	Ketone Salt Diet (CT_KS)	Control Diet (MetS_SD)	Ketogenic Diet (MetS_KD)	Ketone Salt Diet (MetS_KS)
Body Weight (g)	494 (39)	487 (30)	493 (22)	641 (40)^****^	722 (58)^++^	607 (56)
Mes. Fat (g/tibia(mm)^3^)	6.1 (1.0)	7.0 (1.5)	4.8 (1.5)	17.0 (1.3)^****^	21.8 (5.5)^++^	18.1 (2.4)
Glucose Levels (mg/dL)	102 (15)	82 (13)	93 (7)	370 (73)^****^	250 (60)^++++^	238 (70)^++++^
Fasting Glucose (mg/dL)	86 (5)	83 (7)	84 (4)	282 (86)^****^	205 (54)^++^	212 (35)^+^
IRT (AUC)	6 102 (560)	5 743 (368)	6 588 (1 354)	29 868 (7 772)^****^	24 575 (7 456)	25 475 (5 983)
OGTT (AUC)	12 884 (542)	15 045 (1 119)	13 075 (856)	44 144 (5 679)^****^	37 774 (8 004)	45 903 (5 644)
FGF 21 Levels (pg/mL)	193 (126)	1 577 (723)	180 (43)	1 188 (596)	6 566 (6 994)^++^	1 247 (416)
Leptin Levels	182 (129)	484 (242)	313 (128)	979 (687)	4 471 (3 149)^++++^	1 090 (1 093)
VO_2_max(ml/min/kg^0.75^)	38.96 (1.82)	37.23 (2.01)	36.73 (1.36)	17.05 (2.04)^****^	15.44 (2.90)	16.32 (2.95)
VCO_2_max(ml/min/kg^0.75^)	36.13 (1.79)	32.61 (2.09)	34.49 (1.40)	17.11 (1.68)^****^	15.11 (2.23)	17.12 (2.54)
EE (kcal/day/kg^0.75^)	278 (13)	262 (15)	263 (10)	124 (14)^****^	112 (20)	120 (21)
Distance (m)	525 (36)	487 (117)	514 (79)	100 (40)^****^	112 (39)	87 (40)
Ketone levels (mmol/L)	0.66 (0.17)	2.25 (0.64)^****^	0.72 (0.09)	0.59 (0.24)	2.06 (0.82)^++++^	1.01 (0.68)
Ketones Tolerance Test	131 (11)	491 (99)^****^	130 (26)	218 (58)	642 (262)^++++^	185 (70)

Data are presented as the mean (standard deviation). Significant differences (*p* < 0.05) compared to CT_SD are indicated as ^*^ and compared to MetS_SD are indicated as ^+^, by two-way analysis of variance (ANOVA) with Šídák’s multiple comparisons test: *^/+^: *p* < 0.05; **^/++^: *p* < 0.01; ***^/+++^: *p* < 0.001; ****^/++++^: *p* < 0.0001. CT, control phenotype; MetS, metabolic syndrome phenotype; SD, standard/control diet; KD, ketogenic diet; KS, standard/control diet with ketone salts supplementation; Mes. Fat, mesenteric fat; IRT, insulin resistance test; AUC, area under the curve; OGTT, oral glucose tolerance test; FGF 21, fibroblast growth factor 21; VO_2_max, maximum oxygen consumption; VCO_2_max, maximum carbon dioxide production; EE, energy expenditure

### Gut microbiota

**Fig. 1:**
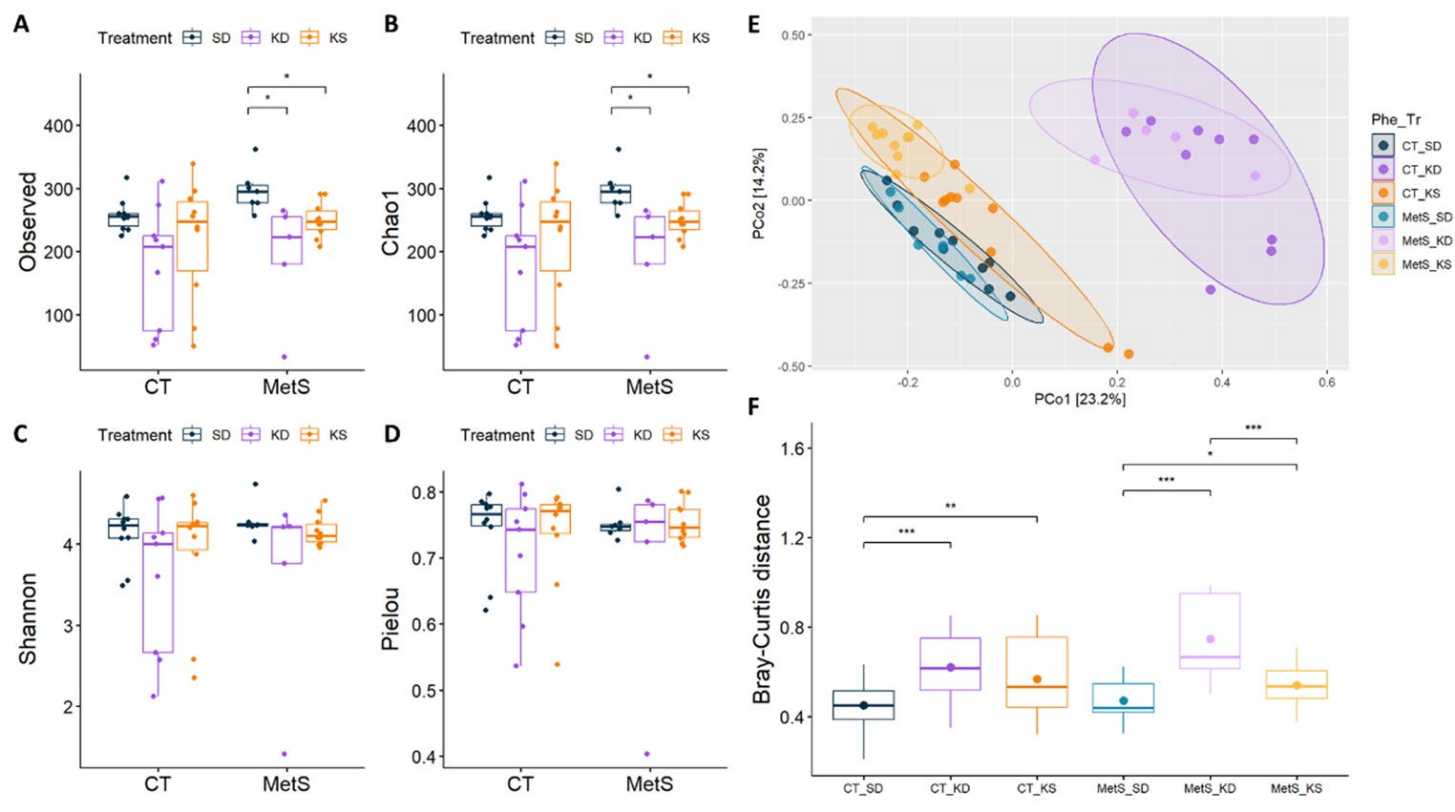
Ketogenic diet and ketone salts change gut microbiota composition. Left: α-diversity plots of fecal microbiota in ZSF1 rats according to phenotype and dietary interventions, Species Richness (Observed and Chao1 indexes, **A and B**), Species Diversity (Shannon index, **C**), and Species Evenness (Pielou index, **D**). Right: β-diversity with principal coordinates analyses (PCoA, **E**) of ZSF1 rat fecal microbiota by phenotype and dietary interventions: each point represents one individual, and each circle represents a microbial community; and the Bray-Curtis distance plot (**F**). Significant differences between the groups are indicated as *: *p* < 0.05 by Wilcoxon Rank Sum Test. CT, control phenotype; MetS, metabolic syndrome phenotype; SD, standard/control diet; KD, ketogenic diet; KS, standard/control diet with ketone salts supplementation

**Fig. 2:**
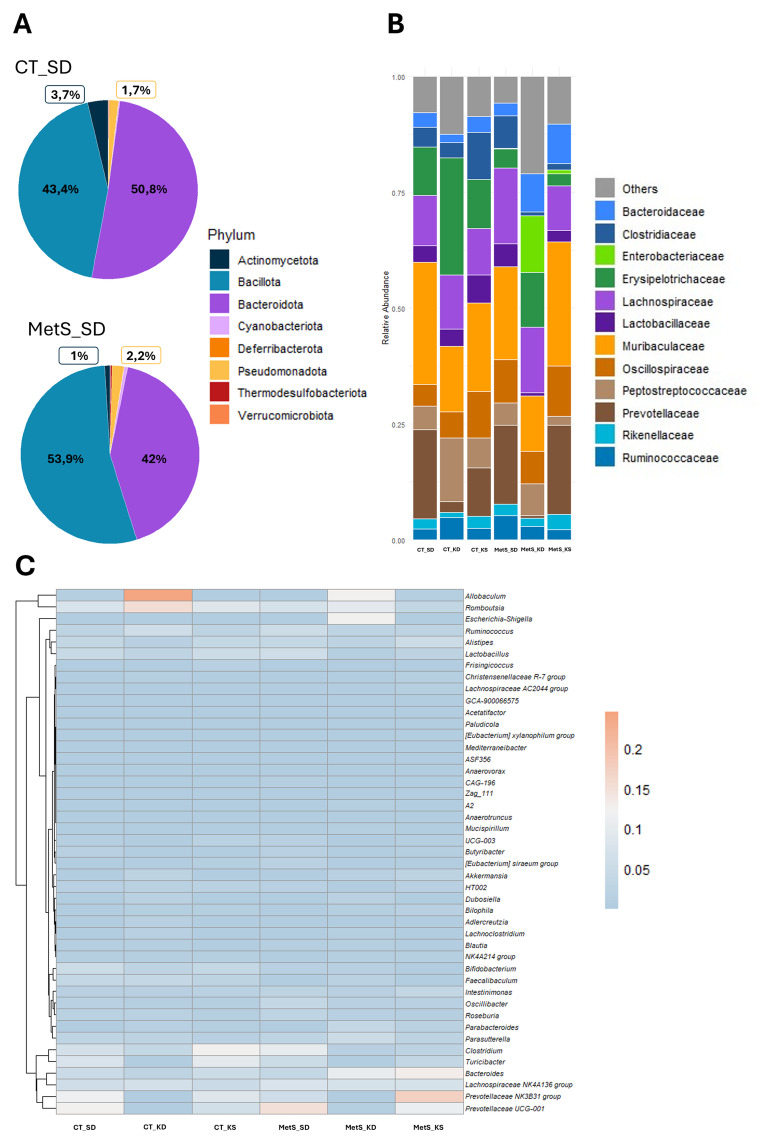
Relative abundances of the bacteria at several taxonomic levels. Donut chart of the 8 dominant phyla according to both phenotypes with control diet (**A**), stacked plot bar of the 12 dominant family levels (**B**) and heatmap of the top genera (**C**) for both phenotypes and the three dietary interventions. CT, control phenotype; MetS, metabolic syndrome phenotype; SD, standard/control diet; KD, ketogenic diet; KS, standard/control diet with ketone salts supplementation. Relative abundance plots were used for descriptive visualization of community composition, while differential abundance was formally tested using ANCOM-BC with FDR correction

Based on gDNA quality thresholds and sequencing depth, nine samples were excluded, leaving 51 samples (CT_SD = 10, CT_KD = 9, CT_KS = 10, MetS_SD = 7, MetS_KD = 5, MetS_KS = 10). Some differences were found when the diversity and composition between groups were compared (Fig. 1). Although no differences were found when comparing the two phenotypes, CT_SD and MetS_SD, when assessing the dietary interventions, both the ketogenic diet and ketone salts supplementation were associated with lower species richness (*p* = 0.049, MetS_SD vs. MetS_KD and MetS_SD vs. MetS_KS, Fig. 1A and B and Table S5). Species diversity and species evenness showed no differences when comparing both phenotypes and dietary interventions (Fig. 1C and D). Although phenotype alone did not alter α-diversity, both KD and KS reduced species richness compared with MetS_SD (Observed and Chao1, *p* = 0.049). Shannon diversity and evenness remained unchanged. Assessing the overall composition of the gut microbiota across samples revealed no significant differences between the fecal communities of control and MetS rats (Fig. 1E and F). However, principal coordinate analysis (PCoA) and the Wilcoxon Rank Sum Test based on Bray-Curtis distance showed significant differences in microbiota profile among the six groups (Fig. 1E and F and Table S6). In short, both KD and KS change the gut microbiota community of the rats (Fig. 1E, F and Table S6). A multivariable PERMANOVA demonstrated that both diet and phenotype significantly influenced gut microbiota composition (Table S7). The profiles of the fecal matter showed some differences at higher and lower taxonomic levels. The results showed that, in comparison to controls, MetS rats had a higher Bacillota-to-Bacteroidota ratio and a lower relative abundance of the phylum Actinomycetota (Figs. 2A and S3A). At the family level, MetS_SD rats had higher levels of Ruminococcaceae, Lachnospiraceae, Oscillospiraceae, and Clostridiaceae and lower levels of Muribaculaceae and Erysipelotrichaceae compared to CT_SD (Fig. 2B). At the genus level, MetS_SD rats presented lower relative abundance in *Allobaculum*, *Bifidobacterium*, Prevotellaceae NK3B31 group, and *Turicibacter* and higher relative abundance in *Clostridium*, Lachnospiraceae NK4A136 group, *Lactobacillus*, Prevotellaceae UCG-001, and *Ruminococcus* compared to the CT_SD group (Figs. 2C and S3).

Considering the influence of dietary interventions, changes were found on several levels in the MetS phenotype. Compared to the control diet group (MetS_SD), the ketogenic diet (MetS_KD) presented lower Bacillota levels and a higher relative abundance of Pseudomonadota (Figure S3). At the family level, KD presented higher Erysipelotrichaceae, Peptostreptococcaceae, and Bacteroidaceae, while lower Prevotellaceae, Clostridiaceae, Lactobacillaceae, and Ruminococcaceae (MetS_KD, Fig. 2B). At the genus level, KD showed higher levels in *Romboutsia*, *Allobaculum*, and *Bacteroides*, and lower levels in Prevotellaceae NK3B31 group, Prevotellaceae UCG-001, *Clostridium*, *Lactobacillus*, and *Ruminococcus* in MetS phenotype (MetS_KD, Figs. 2C and S3). Interestingly, the intervention with KS showed a similar profile as SD in the control phenotype (Figs. 2 and S3). Importantly, while these compositional patterns were evident in relative abundance profiles (Figs. 2 and S3), formal differential abundance testing using ANCOM-BC with Benjamini–Hochberg correction did not identify statistically significant differences between groups after multiple-testing adjustment (Tables S8–S10). These results indicate that the observed shifts reflect overall community-level trends rather than robust changes in individual taxa.

### Histology of microvilli

**Fig. 3:**
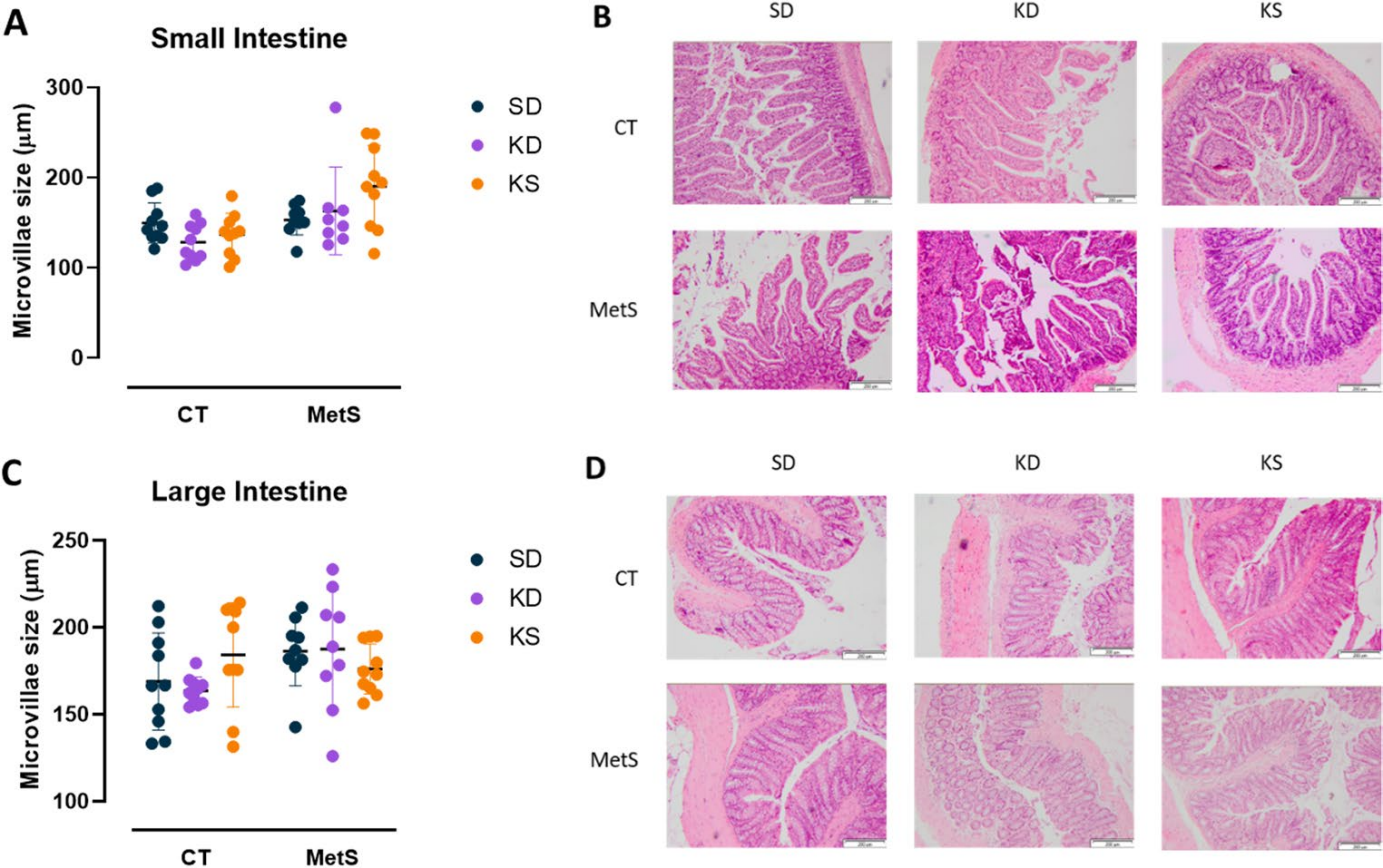
Effects of nutritional intervention on intestinal morphology in lean and obese rats. CT, control phenotype; MetS, metabolic syndrome phenotype; SD, standard/control diet; KD, ketogenic diet; KS, standard/control diet with ketone salts supplementation

To evaluate the effects of the different nutritional interventions on intestinal mucosal morphology, HE-stained small and large intestine sections were examined (Fig. 3). Histological analysis indicated no differences between phenotypes or interventions. The intestinal villi were packed and intact in both phenotypes and the three dietary interventions. In addition, the microvilli size showed no significant differences between groups.

## Discussion

In this work, we examined the metabolic function and gut microbiota alterations that occur in a model of MetS, the obese ZSF1 rat, as well as the impact of a ketogenic diet (KD) and ketone salt (KS) supplementation under control and dysmetabolic conditions. Our findings confirm that MetS induces alteration in glucose metabolism and triggers a subtle shift in gut microbiota composition; however, the effects of KD and KS on the gut microbiota were distinct rather than uniformly beneficial. KD produced substantial changes in microbial diversity and taxonomic profile, whereas KS preserved a community composition more similar to the standard diet.

Prior research has linked the gut microbiota composition to metabolic impairment in MetS [31], and dietary patterns, including KD, have been shown to influence microbial diversity and host metabolism [16, 32]. In our study, α-diversity was reduced most prominently in KD-fed rats, consistent with previous reports of diet-induced reductions in microbial richness [33, 34]. KS, in contrast, produced a milder shift and maintained diversity closer to the standard diet. These observations highlight that the mode of achieving ketone elevation, endogenous vs. exogenous, has distinct consequences for gut microbial ecology.

Reduced microbial diversity is frequently observed in obesity and diabetes [35], and our results align with these trends. KD markedly reduced species’ richness. This reduction was consistent across the observed species index, which quantifies the number of distinct species, and the Chao1 index, an estimator of total species richness that accounts for rare species. Previous studies showed that KD led to a decrease in the overall number of gut bacteria in an animal model [32, 36]. This “antimicrobial” effect caused by the diet is explained by the crucial function of the gut microbiota in digesting undigested carbohydrates, which is markedly reduced in the KD. KS, despite inducing modest ketonemia, did not substantially reduce α-diversity, suggesting that exogenous ketones do not replicate the broad ecological pressures imposed by the KD. These distinctions may have clinical relevance if preserving microbial diversity is considered beneficial in metabolic disease (β-diversity).

At the phylum level, the dominant taxa (Bacillota, Bacteroidota, Pseudomonadota, and Actinomycetota) accounted for more than 90% of intestinal microbiota composition, and numerous studies have linked variations in the Bacillota/Bacteroidota (formerly, Firmicutes/Bacteroidetes) ratio to the lean or obese state [37]. Consistent with the current tendency, MetS was associated with an increased Bacillota/Bacteroidota ratio and reduced Actinomycetota, in agreement with patterns commonly reported in metabolic impairment [37, 38]. At the family and genus levels, MetS rats displayed higher Lachnospiraceae and Ruminococcaceae families, consistent with reports associating these groups with metabolic dysfunction (impaired glucose metabolism, hyperglycemia, and diabetes) [39–42]. As a result, alterations in these gut bacteria may trigger an inflammatory response, resulting in metabolic abnormalities. KD significantly reshaped microbial composition, increasing *Romboutsia* and *Bacteroides* while decreasing SCFA-related genera such as *Lactobacillus*. KS intervention resulted in comparatively moderate taxonomic shifts and maintained a community profile resembling a standard diet, suggesting that exogenous ketones influence microbial composition less dramatically than KD.

Our ZSF1 model is an obese leptin-receptor knockout rat [43], a feature that profoundly alters satiety signalling and energy homeostasis. Without a functional leptin receptor, these animals are unable to accurately sense energy sufficiency. Consequently, even when caloric intake decreased, as occurred under KD, energy expenditure remained low, and adipose accumulation persisted. This mutation has implications for endocrine responses. Leptin resistance enhanced FGF-21 levels in KD-fed rats, reflecting a compensatory response to impaired energy sensing rather than dietary efficacy. Current research on the influence of FGF-21 on carbohydrate and lipid metabolism is debatable, although it has been suggested that KD’s potential success in treating obesity through weight loss and improved insulin tolerance may be related to FGF-21 levels [44]. FGF-21 levels have been reported to decrease in obese and type 2 diabetes patients who lose weight, implying that FGF-21 signaling is improved, similar to the impact on insulin resistance [44]. Thus, FGF-21 appears to play a central role in coordinating the response to interventions that stimulate ketogenesis. These model-specific constraints should be considered when extrapolating results to humans.

Given the increasing prevalence of diabetes and MetS, there is an urgent need for alternative types of interventions that minimize and prevent the dysmetabolism associated with these risk factors. Dietary ketosis has demonstrated therapeutic relevance in the prevention, mitigation, and reversal of metabolic abnormalities, as well as their progression to obesity and diabetes [45]. Hence, nutritional ketosis is an important preventative and restorative therapy modality. Both KD and KS reduced fasting glucose, indicating improved basal glycemic control. However, OGTT and IR test results showed no significant improvement, suggesting that enhanced glucose tolerance was not a dominant outcome of either intervention in this model. This discordance between fasting glucose improvements and adiposity gain in KD-fed rats highlights the unique physiology of ZSF1 animals and supports the notion that KD’s metabolic effects depend on intact leptin signalling [46].

Consistent with prior work, MetS rats showed impaired exercise tolerance, a marker for MetS [47]. Neither KD nor KS improved VO_2_max or energy expenditure, although KS maintained values closer to the standard diet. These factors likely decrease energy expenditure and limit mobilization of stored lipids, contributing to fat accumulation despite reduced caloric intake.

Although KD and KS altered adiposity and glucose parameters, we acknowledge that the absence of fecal mass measurements and continuous locomotor activity limits our ability to fully quantify metabolizable energy and energy expenditure. Additionally, the reduced number of sequenced samples for some groups, due to lipid-rich fecal material that impaired gDNA extraction, lowered the statistical power for some pairwise taxon comparisons and necessitates caution in overinterpreting marginally significant differences. We therefore prioritize effect sizes, consistency across methods (ANCOM-BC), and BH-adjusted q-values in our interpretation and suggest that follow-up studies include optimized extraction protocols or larger cohorts to validate taxa-level findings. Second, functional metagenomic predictions (e.g., PICRUSt2 [48] or Tax4Fun2 [49]) were not performed, and therefore our microbiome analysis is limited to taxonomic inferences. Future work should incorporate functional and metabolomic profiling, particularly pathways related to SCFA metabolism and LPS biosynthesis, to clarify the mechanistic links between microbial shifts and host metabolism. Finally, the use of a leptin-receptor-deficient model may limit translational applicability, as responses to KD and exogenous ketones can differ in animals with intact leptin signalling.

To the best of our knowledge, this is the first study directly comparing the metabolic and microbial effects of KD and KS in a MetS model. Our findings show that KD induces marked microbial restructuring and increases adiposity in ZSF1 rats, whereas KS produces milder microbial changes while improving fasting glucose without exacerbating weight gain. At present, our results indicate that supplementing the control diet with exogenous ketone salts warrants further investigation as a complementary or alternative strategy for achieving ketosis in individuals with Mets.

## Conclusion

In conclusion, our study demonstrates that the ketogenic diet and ketone salt supplementation exert markedly different metabolic and microbiota effects in the ZSF1 MetS model. Although both interventions improved fasting glucose levels, KD induced significant weight gain and reduced microbial richness, accompanied by shifts in taxonomic composition. In contrast, KS maintained a microbial profile similar to the standard diet and did not exacerbate adiposity, while still achieving measurable increases in circulating ketones. These findings highlight that the method of inducing ketosis – dietary versus exogenous – carries distinct physiological consequences. KS may therefore represent a more practical and microbiota-preserving strategy for accomplishing ketone-related metabolic benefits in the context of MetS.

## Supplementary Information

Below is the link to the electronic supplementary material.

Supplementary Material 1

## Data Availability

The microbiome raw data of this study is available at NCBI using the BioProject ID PRJNA1303361.
