# Supplementary material for: Metabolic and gut microbiota effects of ketogenic diet and exogenous ketone salts in a rat model of metabolic syndrome
Source: Eur J Nutr. 2026 Apr 28;65(4):124. doi: 10.1007/s00394-026-03967-z (PMC13124902; doi:10.1007/s00394-026-03967-z)
Supplement: Supplementary file 1 — Supplementary Material 1 [file 394_2026_3967_MOESM1_ESM.pdf]

Supplementary material

Table S1. Descriptive post-hoc power estimates for fasting glucose (primary endpoint).

Comparison	n	n	Cohen's d	95% CI for d	Power (α = 0.05)	Power (Bonferroni α = 0.025)
**KD vs SD**	10	10	**0.92**	−1.83 to 0.02	**0.49**	**0.37**
**KS vs SD**	10	10	**0.31**	−0.58 to 1.19	**0.10**	**0.06**

Table S2. Overall Treatment Effect (ANOVA).

Statistic	Value	95% CI	Statistic
**η² (eta squared)**	**0.47**	0.20 to 1.00	**η² (eta squared)**
**ANOVA power (α = 0.05)**	**0.99**	----	**ANOVA power (α = 0.05)**

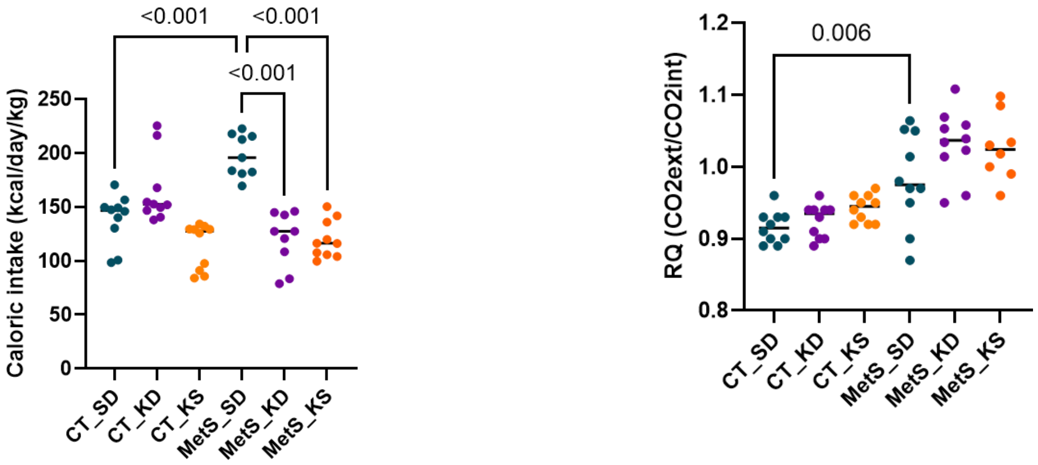

Figure S1. Daily caloric intake (left) and respiratory quotient (RQ) obtained during effort tolerance testing normalized to body weight by the end of the study 23-25 weeks.

Table S3. Ketonemia (blood BHB concentration) at 24 weeks (expressed in mmol/L).

	Control Diet		Ketogenic Diet		Ketone Salt	
	**Mean**	**SD**	**Mean**	**SD**	**Mean**	**SD**
**Control (CT)**	0.60	0.07	2.04	0.82	0.73	0.16
**Metabolic Syndrome (MetS)**	0.60	0.10	1.23	0.25	0.84	0.25

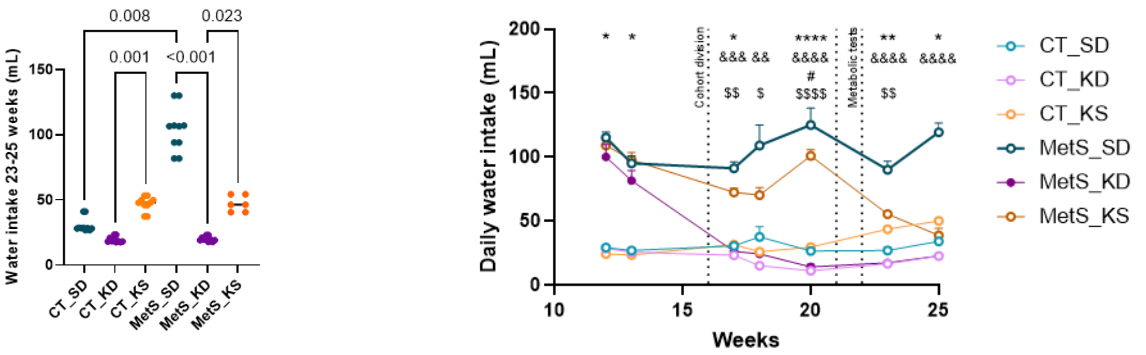

Figure S2 – Daily water intake across the study. Left: Water intake during weejs 23-25. Right: Water intake throughout the study period up to week 25, significant differences are indicated as *CT vs MetS, $^{\&}$MetS vs MetS_KD, $^{\#}$MetS vs MetS_KS, $^{\$}$MetS_KD vs MetS_KS. One symbol: $p<0.05$; two symbols: $p<0.01$; three symbols: $p<0.001$; four symbols: $p<0.0001$. CT, control phenotype; MetS, metabolic syndrome phenotype; SD, standard/control diet; KD, ketogenic diet; KS, standard/control diet with ketone salts supplementation.

Table S4. Estimated daily BHB intake from KS (mg/day per rat).

Period	Group	Water intake (mL/day)	Assumed BW (g)	BHB concentration (g/L)	BHB/day (g/day)
**Weeks 16–23**	Lean	25	430	51.6	~1.29
**Weeks 16–23**	Obese	100	580	17.4	~1.74
**Week 24+**	Lean	45	470	31.3	~1.41
**Week 24+**	Obese	55	590	32.2	~1.77

Table S5 – Alfa-diversity index measure by Wilcoxon Rank Sum Test. CT, control phenotype; MetS, metabolic syndrome phenotype; SD, standard diet; KD, ketogenic diet; KS, standard diet with ketone salts supplementation.

Measure	Phenotype	Comparison	Test Method	P.unadj	P.adj	Significance
Observed	CT	SD - KD	Wilcoxon Rank Sum Test	0.027350610	0.11171491	ns
Observed	CT	SD - KS	Wilcoxon Rank Sum Test	0.762195338	0.92147852	ns
Observed	CT	KD - KS	Wilcoxon Rank Sum Test	0.277511962	0.57023221	ns
Observed	MetS	SD - KD	Wilcoxon Rank Sum Test	0.005050505	0.04943493	*
Observed	MetS	SD - KS	Wilcoxon Rank Sum Test	0.007209261	0.04943493	*
Observed	MetS	KD - KS	Wilcoxon Rank Sum Test	0.296995942	0.57023221	ns
Chao1	CT	SD - KD	Wilcoxon Rank Sum Test	0.027350610	0.11171491	ns
Chao1	CT	SD - KS	Wilcoxon Rank Sum Test	0.762195338	0.92147852	ns
Chao1	CT	KD - KS	Wilcoxon Rank Sum Test	0.277511962	0.57023221	ns
Chao1	MetS	SD - KD	Wilcoxon Rank Sum Test	0.005050505	0.04943493	*
Chao1	MetS	SD - KS	Wilcoxon Rank Sum Test	0.007209261	0.04943493	*
Chao1	MetS	KD - KS	Wilcoxon Rank Sum Test	0.296995942	0.57023221	ns
Shannon	CT	SD - KD	Wilcoxon Rank Sum Test	0.242806729	0.57023221	ns
Shannon	CT	SD - KS	Wilcoxon Rank Sum Test	0.739364351	0.92147852	ns
Shannon	CT	KD - KS	Wilcoxon Rank Sum Test	0.356232003	0.65765908	ns
Shannon	MetS	SD - KD	Wilcoxon Rank Sum Test	0.202020202	0.57023221	ns
Shannon	MetS	SD - KS	Wilcoxon Rank Sum Test	0.229535171	0.57023221	ns
Shannon	MetS	KD - KS	Wilcoxon Rank Sum Test	0.767898768	0.92147852	ns
Pielou	CT	SD - KD	Wilcoxon Rank Sum Test	0.400181861	0.66236998	ns
Pielou	CT	SD - KS	Wilcoxon Rank Sum Test	0.970512460	0.99116166	ns
Pielou	CT	KD - KS	Wilcoxon Rank Sum Test	0.496698348	0.79471736	ns
Pielou	MetS	SD - KD	Wilcoxon Rank Sum Test	1.000000000	1.00000000	ns
Pielou	MetS	SD - KS	Wilcoxon Rank Sum Test	0.886774990	0.94589332	ns
Pielou	MetS	KD - KS	Wilcoxon Rank Sum Test	0.953046953	0.99116166	ns

Table S6 – Beta-diversity index measure by Wilcoxon Rank Sum Test

Comparison	Test Method	P.unadj	P.adj	Significance
CT_SD - CT_KD	Wilcoxon Rank Sum Test	1.340599e-07	2.010898e-06	***
CT_SD - CT_KS	Wilcoxon Rank Sum Test	2.772518e-03	6.931294e-03	**
CT_SD - MetS_SD	Wilcoxon Rank Sum Test	5.567724e-01	6.424297e-01	ns
CT_SD - MetS_KD	Wilcoxon Rank Sum Test	2.482029e-04	9.307608e-04	***
CT_SD - MetS_KS	Wilcoxon Rank Sum Test	2.502027e-05	1.876520e-04	***
CT_KD - CT_KS	Wilcoxon Rank Sum Test	1.300494e-01	1.773401e-01	ns
CT_KD - MetS_SD	Wilcoxon Rank Sum Test	1.261972e-04	6.309861e-04	***
CT_KD - MetS_KD	Wilcoxon Rank Sum Test	8.198756e-01	8.784382e-01	ns
CT_KD - MetS_KS	Wilcoxon Rank Sum Test	5.491231e-03	1.176692e-02	*
CT_KS - MetS_SD	Wilcoxon Rank Sum Test	4.842619e-02	8.071032e-02	ns
CT_KS - MetS_KD	Wilcoxon Rank Sum Test	3.819289e-01	4.774112e-01	ns
CT_KS - MetS_KS	Wilcoxon Rank Sum Test	9.871879e-01	9.871879e-01	ns
MetS_SD - MetS_KD	Wilcoxon Rank Sum Test	2.013445e-03	6.040336e-03	**
MetS_SD - MetS_KS	Wilcoxon Rank Sum Test	9.665934e-03	1.812363e-02	*
MetS_KD - MetS_KS	Wilcoxon Rank Sum Test	5.803272e-02	8.704908e-02	ns

Table S7. PERMANOVA (adonis2) using Bray–Curtis distances for both Treatment and Phenotype.

	Df	SumOfSqs	R2	F	Pr(>F)
**Treatment**	2	3.2895	0.26937	9.4624	0.001
**Phenotype**	1	0.6627	0.05426	3.8123	0.002
**Residual**	47	8.1696	0.66898	----	----
**Total**	50	12.2119	1.00000	----	----

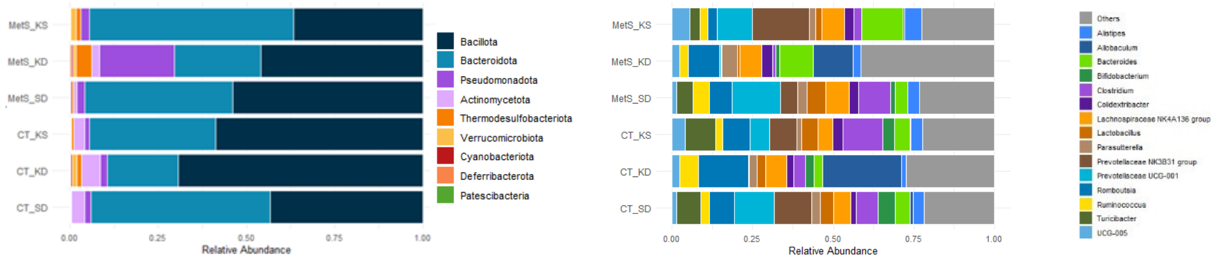

Figure S3. Stacked plot bar of relative abundances of the 9 dominant phylum level (**left**) and the 15 top genus (**right**) for both phenotypes and the three dietary interventions. CT, control phenotype; MetS, metabolic syndrome phenotype; SD, standard diet; KD, ketogenic diet; KS, standard diet with ketone salts supplementation. Relative abundance plots were used for descriptive visualization of community composition, while differential abundance was formally tested using ANCOM-BC with FDR correction.

Table S8.1 – Differentially abundant Phylum identified using ANCOM-BC. Log fold changes (LFCs) and 95% confidence intervals are shown for each contrast. Multiple testing was controlled using the Benjamini–Hochberg false discovery rate (FDR); adjusted p-values (P_adj) are reported.

Taxon	Comparison	LFC	CI_low	CI_high	P_unadj	P_adj	Significance
Actinomycetota	CT_SDvsMetS_SD	-1,4918	-2,5121	-0,4716	0,0064	0,0508	ns
Bacillota	CT_SDvsMetS_SD	0,2589	-0,1007	0,6185	0,1651	0,3303	ns
Bacteroidota	CT_SDvsMetS_SD	-0,1583	-0,5956	0,2790	0,4817	0,5505	ns
Cyanobacteriota	CT_SDvsMetS_SD	1,0737	-0,0552	2,2027	0,0698	0,2793	ns
Deferribacterota	CT_SDvsMetS_SD	0,6192	-0,8199	2,0582	0,4053	0,5505	ns
Pseudomonadota	CT_SDvsMetS_SD	0,2395	-0,3974	0,8764	0,4650	0,5505	ns
Thermodesulfobacteriota	CT_SDvsMetS_SD	0,2159	-1,0896	1,5214	0,7474	0,7474	ns
Verrucomicrobiota	CT_SDvsMetS_SD	-0,5871	-1,3624	0,1883	0,1449	0,3303	ns

Table S8.2 – Differentially abundant Phylum identified using ANCOM-BC. Log fold changes (LFCs) and 95% confidence intervals are shown for each contrast. Multiple testing was controlled using the Benjamini–Hochberg false discovery rate (FDR); adjusted p-values (P_adj) are reported.

Taxon	Comparison	LFC	CI_low	CI_high	P_unadj	P_adj	Significance
Actinomycetota	MetS_SDvsMetS_KD	-0,8027	-2,0969	0,4915	0,2306	0,3075	ns
Bacillota	MetS_SDvsMetS_KD	-0,3003	-1,3423	0,7418	0,5750	0,6572	ns
Bacteroidota	MetS_SDvsMetS_KD	-0,8827	-1,6806	-0,0849	0,0354	0,0727	ns
Cyanobacteriota	MetS_SDvsMetS_KD	0,3143	-1,3522	1,9809	0,7136	0,7136	ns
Deferribacterota	MetS_SDvsMetS_KD	2,5107	0,7247	4,2967	0,0096	0,0384	*
Pseudomonadota	MetS_SDvsMetS_KD	1,6608	0,0312	3,2904	0,0518	0,0829	ns
Thermodesulfobacteriota	MetS_SDvsMetS_KD	2,7536	0,8907	4,6166	0,0059	0,0384	*
Verrucomicrobiota	MetS_SDvsMetS_KD	1,6366	0,1508	3,1225	0,0363	0,0727	ns

Table S8.3 – Differentially abundant Phylum identified using ANCOM-BC. Log fold changes (LFCs) and 95% confidence intervals are shown for each contrast. Multiple testing was controlled using the Benjamini–Hochberg false discovery rate (FDR); adjusted p-values (P_adj) are reported.

Taxon	Comparison	LFC	CI_low	CI_high	P_unadj	P_adj	Significance
Actinomycetota	MetS_SDvsMetS_KS	-4,6017	-5,3834	-3,8199	0,0000	0,0000	***
Bacillota	MetS_SDvsMetS_KS	-0,3589	-0,7153	-0,0026	0,0545	0,1090	ns
Bacteroidota	MetS_SDvsMetS_KS	-0,0468	-0,4737	0,3801	0,8309	0,9960	ns
Cyanobacteriota	MetS_SDvsMetS_KS	-0,9790	-2,2190	0,2609	0,1298	0,2077	ns
Deferribacterota	MetS_SDvsMetS_KS	-0,0659	-1,3666	1,2348	0,9215	0,9960	ns
Pseudomonadota	MetS_SDvsMetS_KS	0,0019	-0,7137	0,7174	0,9960	0,9960	ns
Thermodesulfobacteriota	MetS_SDvsMetS_KS	1,9689	1,0105	2,9272	0,0002	0,0009	***
Verrucomicrobiota	MetS_SDvsMetS_KS	1,6407	0,4984	2,7829	0,0073	0,0194	*

Table S9.1 – Differentially abundant Family identified using ANCOM-BC. Log fold changes (LFCs) and 95% confidence intervals are shown for each contrast. Multiple testing was controlled using the Benjamini–Hochberg false discovery rate (FDR); adjusted p-values (P_adj) are reported.

Taxon	Comparison	LFC	CI_low	CI_high	P_unadj	P_adj	Significance
Bifidobacteriaceae	CT_SDvsMetS_SD	-1,7507	-3,4038	-0,0975	0,0446	0,3534	ns
Eggerthellaceae	CT_SDvsMetS_SD	-1,1790	-2,4508	0,0928	0,0778	0,3585	ns
Bacteria_Bacillota_Bacilli_Erysipelotrichales_	CT_SDvsMetS_SD	1,1832	-0,8000	3,1663	0,2605	0,5938	ns
Erysipelatoclostridiaceae	CT_SDvsMetS_SD	0,3131	-1,4865	2,1126	0,7365	0,8828	ns
Erysipelotrichaceae	CT_SDvsMetS_SD	-0,9430	-1,8511	-0,0348	0,0478	0,3534	ns
Bacteria_Bacillota_Bacilli_Izemoplasmatales_	CT_SDvsMetS_SD	0,8186	-0,8879	2,5252	0,3565	0,5941	ns
Lactobacillaceae	CT_SDvsMetS_SD	0,3817	-0,3147	1,0782	0,2884	0,5938	ns
Bacteria_Bacillota_Bacilli_RF39_	CT_SDvsMetS_SD	0,5623	-0,7517	1,8763	0,4067	0,6128	ns
Bacteria_Bacillota_Clostridia___	CT_SDvsMetS_SD	1,5175	-0,1831	3,2182	0,0926	0,3585	ns

Christensenellaceae	CT_SDvsMetS_SD	-0,5583	-1,8613	0,7446	0,4061	0,6128	ns
Bacteria_Bacillota_Clostridia_Clostridia UCG-014_	CT_SDvsMetS_SD	0,4532	-0,4917	1,3980	0,3524	0,5941	ns
Bacteria_Bacillota_Clostridia_Clostridia vadinBB60 group_	CT_SDvsMetS_SD	1,4153	0,0908	2,7398	0,0431	0,3534	ns
Clostridiaceae	CT_SDvsMetS_SD	0,4658	-0,5008	1,4324	0,3499	0,5941	ns
Lachnospiraceae	CT_SDvsMetS_SD	0,4946	-0,0869	1,0760	0,1024	0,3585	ns
Monoglobaceae	CT_SDvsMetS_SD	0,2510	-1,2694	1,7714	0,7484	0,8828	ns
[Eubacterium] coprostanoligenes group	CT_SDvsMetS_SD	-0,6068	-1,7747	0,5612	0,3144	0,5941	ns
Butyricicoccaceae	CT_SDvsMetS_SD	0,9154	-0,3210	2,1518	0,1547	0,4513	ns
Oscillospiraceae	CT_SDvsMetS_SD	0,6889	0,0174	1,3603	0,0505	0,3534	ns
Ruminococcaceae	CT_SDvsMetS_SD	0,7664	0,0389	1,4939	0,0449	0,3534	ns
UCG-010	CT_SDvsMetS_SD	1,4086	-0,0240	2,8412	0,0626	0,3585	ns
Peptococcaceae	CT_SDvsMetS_SD	-0,5859	-1,5942	0,4225	0,2616	0,5938	ns
Anaerovoracaceae	CT_SDvsMetS_SD	-0,5211	-1,8221	0,7799	0,4377	0,6128	ns
Peptostreptococcaceae	CT_SDvsMetS_SD	-0,0265	-0,6344	0,5815	0,9323	0,9323	ns
Bacteroidaceae	CT_SDvsMetS_SD	-0,0906	-0,8767	0,6955	0,8223	0,9068	ns
Muribaculaceae	CT_SDvsMetS_SD	-0,2496	-0,9261	0,4270	0,4735	0,6374	ns
Prevotellaceae	CT_SDvsMetS_SD	0,0483	-0,8722	0,9688	0,9186	0,9323	ns
Rikenellaceae	CT_SDvsMetS_SD	0,1066	-0,8554	1,0687	0,8291	0,9068	ns
Tannerellaceae	CT_SDvsMetS_SD	-0,0917	-1,0706	0,8872	0,8552	0,9071	ns
Gastranaerophilaceae	CT_SDvsMetS_SD	1,1486	-0,1713	2,4685	0,0965	0,3585	ns
Deferribacteraceae	CT_SDvsMetS_SD	0,6192	-0,8669	2,1053	0,4202	0,6128	ns
Bacteria_Pseudomonadota_Alphaproteobacteria_Rhodospirillales_	CT_SDvsMetS_SD	1,0502	-0,3507	2,4511	0,1512	0,4513	ns
Sutterellaceae	CT_SDvsMetS_SD	0,1667	-0,7691	1,1024	0,7287	0,8828	ns
Enterobacteriaceae	CT_SDvsMetS_SD	0,9274	-0,6950	2,5497	0,2728	0,5938	ns
Desulfovibrionaceae	CT_SDvsMetS_SD	0,2159	-1,1413	1,5731	0,7567	0,8828	ns
Akkermansiaceae	CT_SDvsMetS_SD	-0,5871	-1,4467	0,2725	0,1876	0,5050	ns

Table S9.2 – Differentially abundant Family identified using ANCOM-BC. Log fold changes (LFCs) and 95% confidence intervals are shown for each contrast. Multiple testing was controlled using the Benjamini–Hochberg false discovery rate (FDR); adjusted p-values (P_adj) are reported.

Taxon	Comparison	LFC	CI_low	CI_high	P_unadj	P_adj	Significance
Bifidobacteriaceae	MetS_SDvsMetS_KD	-1,4237	-2,9926	0,1452	0,0831	0,2035	ns
Eggerthellaceae	MetS_SDvsMetS_KD	0,3979	-1,3223	2,1182	0,6531	0,7665	ns
Bacteria_Bacillota_Bacilli_Erysipelotrichales_	MetS_SDvsMetS_KD	2,3528	0,1187	4,5870	0,0568	0,1528	ns
Erysipelatoclostridiaceae	MetS_SDvsMetS_KD	1,2493	-1,0602	3,5587	0,3011	0,5181	ns
Erysipelotrichaceae	MetS_SDvsMetS_KD	-0,2803	-1,6082	1,0475	0,6810	0,7688	ns
Bacteria_Bacillota_Bacilli_Izemoplasmatales_	MetS_SDvsMetS_KD	1,6274	-0,3275	3,5823	0,1158	0,2533	ns
Lactobacillaceae	MetS_SDvsMetS_KD	-2,1643	-3,4657	-0,8628	0,0021	0,0186	*
Bacteria_Bacillota_Bacilli_RF39_	MetS_SDvsMetS_KD	-0,9019	-2,5583	0,7545	0,2925	0,5181	ns
Bacteria_Bacillota_Clostridia___	MetS_SDvsMetS_KD	2,3445	0,0816	4,6075	0,0531	0,1528	ns
Christensenellaceae	MetS_SDvsMetS_KD	0,3526	-1,1845	1,8898	0,6555	0,7665	ns
Bacteria_Bacillota_Clostridia_Clostridia UCG-014_	MetS_SDvsMetS_KD	-1,2547	-2,3709	-0,1384	0,0330	0,1309	ns
Bacteria_Bacillota_Clostridia_Clostridia vadinBB60 group_	MetS_SDvsMetS_KD	-0,0881	-1,5937	1,4175	0,9093	0,9361	ns
Clostridiaceae	MetS_SDvsMetS_KD	-1,9878	-3,1011	-0,8745	0,0011	0,0124	*
Lachnospiraceae	MetS_SDvsMetS_KD	-0,3649	-1,8892	1,1594	0,6412	0,7665	ns
Monoglobaceae	MetS_SDvsMetS_KD	0,7278	-0,9000	2,3556	0,3874	0,5895	ns
[Eubacterium] coprostanoligenes group	MetS_SDvsMetS_KD	-1,2804	-2,4714	-0,0894	0,0411	0,1309	ns
Butyricicoccaceae	MetS_SDvsMetS_KD	-0,8446	-2,2252	0,5360	0,2378	0,4623	ns
Oscillospiraceae	MetS_SDvsMetS_KD	0,3486	-0,5759	1,2731	0,4638	0,6764	ns
Ruminococcaceae	MetS_SDvsMetS_KD	-1,1860	-3,4535	1,0815	0,3109	0,5181	ns
UCG-010	MetS_SDvsMetS_KD	1,7311	0,1652	3,2971	0,0376	0,1309	ns
Peptococcaceae	MetS_SDvsMetS_KD	-0,1726	-1,4102	1,0650	0,7860	0,8336	ns

Anaerovoracaceae	MetS_SDvsMetS_KD	-0,2231	-1,7407	1,2946	0,7750	0,8336	ns
Peptostreptococcaceae	MetS_SDvsMetS_KD	0,0204	-0,9993	1,0401	0,9689	0,9689	ns
Bacteroidaceae	MetS_SDvsMetS_KD	0,5064	-0,9948	2,0076	0,5120	0,7169	ns
Muribaculaceae	MetS_SDvsMetS_KD	-0,7964	-1,6886	0,0959	0,0872	0,2035	ns
Prevotellaceae	MetS_SDvsMetS_KD	-3,2953	-4,3085	-2,2821	0,0000	0,0000	***
Rikenellaceae	MetS_SDvsMetS_KD	-0,6239	-2,0210	0,7731	0,3864	0,5895	ns
Tannerellaceae	MetS_SDvsMetS_KD	1,9451	0,6702	3,2200	0,0047	0,0329	*
Gastranaerophilaceae	MetS_SDvsMetS_KD	0,4110	-1,3883	2,2104	0,6570	0,7665	ns
Deferribacteraceae	MetS_SDvsMetS_KD	2,5107	0,6995	4,3218	0,0105	0,0527	ns
Bacteria_Pseudomonadota_Alphaprot eobacteria_Rhodospirillales_	MetS_SDvsMetS_KD	3,1186	1,4438	4,7934	0,0009	0,0124	*
Sutterellaceae	MetS_SDvsMetS_KD	0,4466	-0,9878	1,8811	0,5449	0,7335	ns
Enterobacteriaceae	MetS_SDvsMetS_KD	2,5397	-0,7958	5,8752	0,1476	0,3040	ns
Desulfovibrionaceae	MetS_SDvsMetS_KD	2,7536	0,8665	4,6407	0,0065	0,0380	*
Akkermansiaceae	MetS_SDvsMetS_KD	1,6366	0,1206	3,1527	0,0400	0,1309	ns

Table S9.3 – Differentially abundant Family identified using ANCOM-BC. Log fold changes (LFCs) and 95% confidence intervals are shown for each contrast. Multiple testing was controlled using the Benjamini–Hochberg false discovery rate (FDR); adjusted p-values (P_adj) are reported.

Taxon	Comparison	LFC	CI_low	CI_high	P_unadj	P_adj	Significance
Bifidobacteriaceae	MetS_SDvsMetS_KS	-5,0994	-6,1870	-4,0118	0,0000	0,0000	***
Eggerthellaceae	MetS_SDvsMetS_KS	-1,6754	-2,8788	-0,4719	0,0099	0,0474	*
Bacteria_Bacillota_Bacilli_Erysipelotr ichales_	MetS_SDvsMetS_KS	2,0102	0,0859	3,9344	0,0585	0,1904	ns
Erysipelatoclostridiaceae	MetS_SDvsMetS_KS	1,8110	0,0213	3,6007	0,0606	0,1904	ns
Erysipelotrichaceae	MetS_SDvsMetS_KS	-2,0624	-3,1623	-0,9625	0,0006	0,0074	**
Bacteria_Bacillota_Bacilli_Izemoplas matales_	MetS_SDvsMetS_KS	0,0455	-1,5658	1,6569	0,9563	0,9844	ns
Lactobacillaceae	MetS_SDvsMetS_KS	-0,5954	-1,3009	0,1100	0,1050	0,2626	ns

Bacteria_Bacillota_Bacilli_RF39_	MetS_SDvsMetS_KS	0,1773	-1,1753	1,5300	0,7986	0,9168	ns
Bacteria_Bacillota_Clostridia___	MetS_SDvsMetS_KS	0,7171	-0,8543	2,2886	0,3796	0,5776	ns
Christensenellaceae	MetS_SDvsMetS_KS	0,8492	-0,2947	1,9931	0,1537	0,3586	ns
Bacteria_Bacillota_Clostridia_Clostridia UCG-014_	MetS_SDvsMetS_KS	0,0080	-1,0352	1,0512	0,9881	0,9881	ns
Bacteria_Bacillota_Clostridia_Clostridia vadinBB60 group_	MetS_SDvsMetS_KS	0,8664	-0,6254	2,3581	0,2623	0,5100	ns
Clostridiaceae	MetS_SDvsMetS_KS	-2,1759	-3,6296	-0,7221	0,0053	0,0307	*
Lachnospiraceae	MetS_SDvsMetS_KS	-0,3513	-0,9874	0,2848	0,2848	0,5157	ns
Monoglobaceae	MetS_SDvsMetS_KS	0,2423	-1,2306	1,7152	0,7492	0,9168	ns
[Eubacterium] coprostanoligenes group	MetS_SDvsMetS_KS	0,6473	-0,5483	1,8429	0,2947	0,5157	ns
Butyricicoccaceae	MetS_SDvsMetS_KS	-0,1989	-1,3486	0,9508	0,7364	0,9168	ns
Oscillospiraceae	MetS_SDvsMetS_KS	0,5863	-0,0905	1,2630	0,0966	0,2601	ns
Ruminococcaceae	MetS_SDvsMetS_KS	-0,2774	-0,9706	0,4157	0,4370	0,6373	ns
UCG-010	MetS_SDvsMetS_KS	0,7289	-0,6748	2,1325	0,3162	0,5270	ns
Peptococcaceae	MetS_SDvsMetS_KS	0,1276	-0,8817	1,1370	0,8055	0,9168	ns
Anaerovoracaceae	MetS_SDvsMetS_KS	0,6072	-0,6118	1,8262	0,3356	0,5340	ns
Peptostreptococcaceae	MetS_SDvsMetS_KS	-1,3855	-2,1757	-0,5953	0,0013	0,0112	*
Bacteroidaceae	MetS_SDvsMetS_KS	0,7692	-0,0278	1,5662	0,0653	0,1904	ns
Muribaculaceae	MetS_SDvsMetS_KS	-0,1865	-0,8604	0,4873	0,5902	0,7945	ns
Prevotellaceae	MetS_SDvsMetS_KS	-0,0369	-0,9262	0,8523	0,9355	0,9844	ns
Rikenellaceae	MetS_SDvsMetS_KS	0,1116	-0,8025	1,0258	0,8120	0,9168	ns
Tannerellaceae	MetS_SDvsMetS_KS	1,1180	0,2217	2,0144	0,0189	0,0734	ns
Gastranaerophilaceae	MetS_SDvsMetS_KS	-0,8969	-2,3026	0,5088	0,2189	0,4508	ns
Deferribacteraceae	MetS_SDvsMetS_KS	-0,0659	-1,4250	1,2932	0,9249	0,9844	ns
Bacteria_Pseudomonadota_Alphaproteobacteria_Rhodospirillales_	MetS_SDvsMetS_KS	0,4611	-1,1523	2,0745	0,5792	0,7945	ns
Sutterellaceae	MetS_SDvsMetS_KS	-0,5888	-1,5138	0,3362	0,2189	0,4508	ns
Enterobacteriaceae	MetS_SDvsMetS_KS	2,8954	1,1839	4,6068	0,0027	0,0189	*

Desulfovibrionaceae	MetS_SDvsMetS_KS	1,9689	0,9326	3,0051	0,0006	0,0074	**
Akkermansiaceae	MetS_SDvsMetS_KS	1,6407	0,4324	2,8490	0,0108	0,0474	*

Table S10.1 – Differentially abundant Genus identified using ANCOM-BC. Log fold changes (LFCs) and 95% confidence intervals are shown for each contrast. Multiple testing was controlled using the Benjamini–Hochberg false discovery rate (FDR); adjusted p-values (P_adj) are reported.

**Taxon**	**Comparison**	**LFC**	**CI_low**	**CI_high**	**P_unadj**	**P_adj**	**Significance**
Bifidobacterium	CT_SDvsMetS_SD	-1,7507	-3,4627	-0,0386	0,0520	0,6638	ns
Adlercreutzia	CT_SDvsMetS_SD	-1,1790	-2,5265	0,1685	0,0952	0,6740	ns
Bacteria_Bacillota_Bacilli_Erysipelotrichales___	CT_SDvsMetS_SD	1,1832	-0,8493	3,2156	0,2718	0,7743	ns
*Bacteria_Bacillota_Bacilli_Erysipelotrichales_Erysipelotrichaceae_*	CT_SDvsMetS_SD	-1,9936	-3,4840	-0,5033	0,0129	0,4693	ns
Allobaculum	CT_SDvsMetS_SD	-1,4689	-3,0234	0,0855	0,0716	0,6740	ns
Dubosiella	CT_SDvsMetS_SD	0,1276	-1,4367	1,6919	0,8740	0,9667	ns
Faecalibaculum	CT_SDvsMetS_SD	-1,8257	-3,1637	-0,4876	0,0109	0,4693	ns
Turicibacter	CT_SDvsMetS_SD	-0,4136	-1,8145	0,9874	0,5665	0,8798	ns
Bacteria_Bacillota_Bacilli_Izemoplasmatales___	CT_SDvsMetS_SD	0,8186	-0,9450	2,5823	0,3720	0,8032	ns
HT002	CT_SDvsMetS_SD	0,1132	-0,8416	1,0680	0,8173	0,9623	ns
Lactobacillus	CT_SDvsMetS_SD	0,4973	-0,3537	1,3482	0,2581	0,7743	ns
Bacteria_Bacillota_Bacilli_RF39___	CT_SDvsMetS_SD	0,5623	-0,8250	1,9497	0,4317	0,8232	ns
Bacteria_Bacillota_Clostridia____	CT_SDvsMetS_SD	1,5175	-0,2404	3,2755	0,1031	0,6740	ns
Christensenellaceae R-7 group	CT_SDvsMetS_SD	-0,5583	-1,9352	0,8186	0,4316	0,8232	ns
Bacteria_Bacillota_Clostridia_Clostridia UCG-014___	CT_SDvsMetS_SD	0,4532	-0,5913	1,4976	0,3998	0,8107	ns
Bacteria_Bacillota_Clostridia_Clostridia vadinBB60 group___	CT_SDvsMetS_SD	1,4153	0,0180	2,8125	0,0546	0,6638	ns
Clostridium	CT_SDvsMetS_SD	0,4658	-0,5983	1,5299	0,3955	0,8107	ns
*Bacteria_Bacillota_Clostridia_Lachnospirales_Lachnospiraceae_*	CT_SDvsMetS_SD	0,4691	-0,4404	1,3786	0,3177	0,7997	ns
[Eubacterium] xylanophilum group	CT_SDvsMetS_SD	1,4974	-0,3512	3,3460	0,1266	0,6740	ns

A2	CT_SDvsMetS_SD	-0,6753	-2,1459	0,7954	0,3741	0,8032	ns
Acetatifactor	CT_SDvsMetS_SD	0,4298	-1,5178	2,3775	0,6705	0,9235	ns
ASF356	CT_SDvsMetS_SD	-0,0219	-1,9617	1,9179	0,9825	0,9825	ns
Blautia	CT_SDvsMetS_SD	0,3260	-0,9972	1,6492	0,6320	0,9046	ns
Butyribacter	CT_SDvsMetS_SD	0,2196	-1,6102	2,0495	0,8159	0,9623	ns
Frisingicoccus	CT_SDvsMetS_SD	1,2283	-0,5515	3,0081	0,1893	0,6910	ns
GCA-900066575	CT_SDvsMetS_SD	-0,1134	-1,8987	1,6719	0,9020	0,9743	ns
Lachnoclostridium	CT_SDvsMetS_SD	0,2044	-1,3509	1,7597	0,7982	0,9623	ns
Lachnospiraceae AC2044 group	CT_SDvsMetS_SD	1,3019	-0,4951	3,0990	0,1675	0,6740	ns
Lachnospiraceae NK4A136 group	CT_SDvsMetS_SD	0,4262	-0,4935	1,3460	0,3688	0,8032	ns
Mediterraneibacter	CT_SDvsMetS_SD	-0,6898	-2,7158	1,3362	0,5141	0,8798	ns
Roseburia	CT_SDvsMetS_SD	0,3831	-0,9977	1,7640	0,5896	0,8831	ns
Monoglobus	CT_SDvsMetS_SD	0,2510	-1,3332	1,8352	0,7582	0,9623	ns
*Bacteria_Bacillota_Clostridia_Oscillospirales_[Eubacterium] coprostanoligenes group_*	CT_SDvsMetS_SD	-0,6068	-1,8566	0,6431	0,3468	0,8032	ns
UCG-009	CT_SDvsMetS_SD	0,0494	-1,4121	1,5108	0,9476	0,9743	ns
*Bacteria_Bacillota_Clostridia_Oscillospirales_O scillospiraceae_*	CT_SDvsMetS_SD	0,7706	-0,4087	1,9500	0,2079	0,7225	ns
Colidextribacter	CT_SDvsMetS_SD	0,6199	-0,4797	1,7194	0,2758	0,7743	ns
Intestinimonas	CT_SDvsMetS_SD	0,7679	-0,2712	1,8070	0,1551	0,6740	ns
NK4A214 group	CT_SDvsMetS_SD	-0,4331	-1,6345	0,7683	0,4840	0,8798	ns
Oscillibacter	CT_SDvsMetS_SD	1,2479	0,1852	2,3106	0,0265	0,6450	ns
UCG-003	CT_SDvsMetS_SD	0,8131	-0,3374	1,9635	0,1739	0,6740	ns
UCG-005	CT_SDvsMetS_SD	0,1203	-0,7973	1,0380	0,7984	0,9623	ns
*Bacteria_Bacillota_Clostridia_Oscillospirales_R uminococcaceae_*	CT_SDvsMetS_SD	0,9332	-0,3915	2,2579	0,1754	0,6740	ns
[Eubacterium] siraeum group	CT_SDvsMetS_SD	0,9098	-0,8348	2,6543	0,3144	0,7997	ns
Acutalibacter	CT_SDvsMetS_SD	0,2305	-1,1548	1,6157	0,7462	0,9623	ns
Anaerofilum	CT_SDvsMetS_SD	-0,7134	-2,9791	1,5524	0,5599	0,8798	ns
Anaerotruncus	CT_SDvsMetS_SD	-0,0768	-1,6060	1,4525	0,9223	0,9743	ns
Fournierella	CT_SDvsMetS_SD	0,0368	-2,2059	2,2794	0,9753	0,9825	ns
Paludicola	CT_SDvsMetS_SD	0,2612	-1,8728	2,3952	0,8148	0,9623	ns

Pygmaiobacter	CT_SDvsMetS_SD	0,5157	-1,4053	2,4367	0,6049	0,8831	ns
Ruminococcus	CT_SDvsMetS_SD	0,6802	-0,2431	1,6035	0,1560	0,6740	ns
Ruthenibacterium	CT_SDvsMetS_SD	-0,3104	-2,3659	1,7451	0,7716	0,9623	ns
*Bacteria_Bacillota_Clostridia_Oscillospirales_UCG-010_*	CT_SDvsMetS_SD	1,4086	-0,0916	2,9087	0,0747	0,6740	ns
*Bacteria_Bacillota_Clostridia_Peptococcales_Peptococcaceae_*	CT_SDvsMetS_SD	-0,8388	-1,9506	0,2730	0,1470	0,6740	ns
Peptococcus	CT_SDvsMetS_SD	0,4188	-0,9222	1,7598	0,5443	0,8798	ns
*Bacteria_Bacillota_Clostridia_Peptostreptococcales-Tissierellales_Anaerovoracaceae_*	CT_SDvsMetS_SD	0,0802	-1,8826	2,0431	0,9371	0,9743	ns
Anaerovorax	CT_SDvsMetS_SD	-0,5412	-2,2953	1,2130	0,5511	0,8798	ns
Romboutsia	CT_SDvsMetS_SD	-0,0265	-0,7800	0,7270	0,9454	0,9743	ns
Bacteroides	CT_SDvsMetS_SD	-0,0906	-0,9940	0,8128	0,8451	0,9639	ns
*Bacteria_Bacteroidota_Bacteroidia_Bacteroidales_Muribaculaceae_*	CT_SDvsMetS_SD	-0,2516	-1,0612	0,5581	0,5456	0,8798	ns
Muribaculum	CT_SDvsMetS_SD	0,4789	-1,3032	2,2611	0,6032	0,8831	ns
Prevotellaceae NK3B31 group	CT_SDvsMetS_SD	-0,7480	-2,3549	0,8590	0,3677	0,8032	ns
Prevotellaceae UCG-001	CT_SDvsMetS_SD	0,7708	-0,5189	2,0604	0,2480	0,7743	ns
Alistipes	CT_SDvsMetS_SD	0,1066	-0,9534	1,1667	0,8446	0,9639	ns
Parabacteroides	CT_SDvsMetS_SD	-0,0917	-1,1671	0,9837	0,8681	0,9667	ns
CAG-196	CT_SDvsMetS_SD	1,8633	0,0768	3,6497	0,0504	0,6638	ns
Zag_111	CT_SDvsMetS_SD	0,4898	-1,0963	2,0759	0,5489	0,8798	ns
Mucispirillum	CT_SDvsMetS_SD	0,6192	-0,9322	2,1705	0,4398	0,8232	ns
Bacteria_Pseudomonadota_Alphaproteobacteria_Rhodospirillales___	CT_SDvsMetS_SD	1,0502	-0,4198	2,5201	0,1708	0,6740	ns
Parasutterella	CT_SDvsMetS_SD	0,1667	-0,8695	1,2029	0,7541	0,9623	ns
Escherichia-Shigella	CT_SDvsMetS_SD	0,9274	-0,7549	2,6097	0,2899	0,7837	ns
*Bacteria_Thermodesulfobacteriota_Desulfovibrionia_Desulfovibrionales_Desulfovibrionaceae_*	CT_SDvsMetS_SD	0,3825	-1,2362	2,0012	0,6459	0,9067	ns
Bilophila	CT_SDvsMetS_SD	-1,0090	-2,4195	0,4016	0,1700	0,6740	ns
Akkermansia	CT_SDvsMetS_SD	-0,5871	-1,5551	0,3809	0,2409	0,7743	ns

Table S10.2 – Differentially abundant Genus identified using ANCOM-BC. Log fold changes (LFCs) and 95% confidence intervals are shown for each contrast. Multiple testing was controlled using the Benjamini–Hochberg false discovery rate (FDR); adjusted p-values (P_adj) are reported.

Taxon	Comparison	LFC	CI_low	CI_high	P_unadj	P_adj	Significance
Bifidobacterium	MetS_SDvsMetS_KD	-1,4237	-3,0759	0,2286	0,0992	0,3449	ns
Adlercreutzia	MetS_SDvsMetS_KD	0,3979	-1,3987	2,1945	0,6669	0,8333	ns
Bacteria_Bacillota_Bacilli_Erysipelotrichales___	MetS_SDvsMetS_KD	2,3528	0,0594	4,6463	0,0627	0,2408	ns
Bacteria_Bacillota_Bacilli_Erysipelotrichales_Erysipelotrichaceae_	MetS_SDvsMetS_KD	-0,8328	-2,7153	1,0496	0,3918	0,6904	ns
Allobaculum	MetS_SDvsMetS_KD	2,9373	1,0912	4,7834	0,0034	0,0356	*
Dubosiella	MetS_SDvsMetS_KD	1,9272	-0,1538	4,0081	0,0792	0,2890	ns
Faecalibaculum	MetS_SDvsMetS_KD	-1,0251	-3,0347	0,9845	0,3236	0,6384	ns
Turicibacter	MetS_SDvsMetS_KD	-5,4207	-6,9206	-3,9208	0,0000	0,0000	***
Bacteria_Bacillota_Bacilli_Izemoplasmatales___	MetS_SDvsMetS_KD	1,6274	-0,3950	3,6498	0,1278	0,4057	ns
HT002	MetS_SDvsMetS_KD	-1,1984	-2,6603	0,2636	0,1153	0,3826	ns
Lactobacillus	MetS_SDvsMetS_KD	-2,2207	-3,5161	-0,9253	0,0016	0,0291	*
Bacteria_Bacillota_Bacilli_RF39___	MetS_SDvsMetS_KD	-0,9019	-2,6374	0,8337	0,3147	0,6384	ns
Bacteria_Bacillota_Clostridia____	MetS_SDvsMetS_KD	2,3445	0,0230	4,6660	0,0589	0,2408	ns
Christensenellaceae R-7 group	MetS_SDvsMetS_KD	0,3341	-1,2822	1,9504	0,6876	0,8366	ns
Bacteria_Bacillota_Clostridia_Clostridia UCG-014___	MetS_SDvsMetS_KD	-1,2547	-2,4853	-0,0240	0,0520	0,2408	ns
Bacteria_Bacillota_Clostridia_Clostridia vadinBB60 group___	MetS_SDvsMetS_KD	-0,0881	-1,6804	1,5042	0,9142	0,9888	ns
Clostridium	MetS_SDvsMetS_KD	-1,9878	-3,2157	-0,7598	0,0027	0,0331	*
Bacteria_Bacillota_Clostridia_Lachnospirales_Lachnospiraceae_	MetS_SDvsMetS_KD	0,2415	-1,0814	1,5644	0,7223	0,8613	ns
[Eubacterium] xylanophilum group	MetS_SDvsMetS_KD	0,1360	-2,0037	2,2757	0,9020	0,9888	ns
A2	MetS_SDvsMetS_KD	0,5333	-1,2327	2,2992	0,5576	0,7692	ns
Acetatifactor	MetS_SDvsMetS_KD	0,6627	-1,5588	2,8842	0,5660	0,7692	ns
ASF356	MetS_SDvsMetS_KD	-0,7440	-2,9649	1,4770	0,5183	0,7692	ns
Blautia	MetS_SDvsMetS_KD	-0,4751	-2,1934	1,2432	0,5911	0,7845	ns

Butyribacter	MetS_SDvsMetS_KD	-2,1715	-4,2057	-0,1372	0,0467	0,2408	ns
Frisingicoccus	MetS_SDvsMetS_KD	1,5179	-0,5150	3,5507	0,1569	0,4098	ns
GCA-900066575	MetS_SDvsMetS_KD	1,0880	-0,9903	3,1662	0,3155	0,6384	ns
Lachnoclostridium	MetS_SDvsMetS_KD	1,9795	0,4081	3,5508	0,0183	0,1335	ns
Lachnospiraceae AC2044 group	MetS_SDvsMetS_KD	1,3461	-0,6002	3,2924	0,1869	0,4575	ns
Lachnospiraceae NK4A136 group	MetS_SDvsMetS_KD	0,4156	-0,7354	1,5666	0,4829	0,7501	ns
Mediterraneibacter	MetS_SDvsMetS_KD	-0,3770	-2,7300	1,9760	0,7576	0,8778	ns
Roseburia	MetS_SDvsMetS_KD	0,0236	-1,4579	1,5052	0,9752	0,9888	ns
Monoglobus	MetS_SDvsMetS_KD	0,7278	-0,9805	2,4361	0,4099	0,6909	ns
Bacteria_Bacillota_Clostridia_Oscillospirales_[Eubacterium] coprostanoligenes group_	MetS_SDvsMetS_KD	-1,2804	-2,5792	0,0185	0,0601	0,2408	ns
UCG-009	MetS_SDvsMetS_KD	0,2237	-1,4692	1,9166	0,7973	0,8954	ns
Bacteria_Bacillota_Clostridia_Oscillospirales_Oscillospiraceae_	MetS_SDvsMetS_KD	-0,6402	-2,0509	0,7705	0,3792	0,6904	ns
Colidextribacter	MetS_SDvsMetS_KD	0,6460	-0,7297	2,0218	0,3629	0,6904	ns
Intestinimonas	MetS_SDvsMetS_KD	0,4145	-0,8277	1,6568	0,5167	0,7692	ns
NK4A214 group	MetS_SDvsMetS_KD	-0,0297	-1,5341	1,4748	0,9694	0,9888	ns
Oscillibacter	MetS_SDvsMetS_KD	0,0600	-1,3822	1,5022	0,9354	0,9888	ns
UCG-003	MetS_SDvsMetS_KD	-0,8074	-2,2400	0,6252	0,2761	0,6298	ns
UCG-005	MetS_SDvsMetS_KD	0,5842	-0,6915	1,8599	0,3743	0,6904	ns
Bacteria_Bacillota_Clostridia_Oscillospirales_Ruminococcaceae_	MetS_SDvsMetS_KD	0,6901	-0,8894	2,2696	0,3972	0,6904	ns
[Eubacterium] siraeum group	MetS_SDvsMetS_KD	-0,5080	-2,4382	1,4222	0,6095	0,7945	ns
Acutalibacter	MetS_SDvsMetS_KD	0,2301	-1,3351	1,7952	0,7749	0,8839	ns
Anaerofilum	MetS_SDvsMetS_KD	-0,5782	-3,1381	1,9817	0,6735	0,8333	ns
Anaerotruncus	MetS_SDvsMetS_KD	1,0089	-0,8442	2,8620	0,2942	0,6384	ns
Fournierella	MetS_SDvsMetS_KD	-0,0829	-2,6165	2,4507	0,9507	0,9888	ns
Paludicola	MetS_SDvsMetS_KD	1,9314	-0,4984	4,3612	0,1475	0,4098	ns
Pygmaiobacter	MetS_SDvsMetS_KD	0,6954	-1,4764	2,8672	0,5377	0,7692	ns
Ruminococcus	MetS_SDvsMetS_KD	-1,8168	-4,2901	0,6565	0,1572	0,4098	ns
Ruthenibacterium	MetS_SDvsMetS_KD	0,0090	-2,3298	2,3479	0,9941	0,9941	ns
Bacteria_Bacillota_Clostridia_Oscillospirales_U	MetS_SDvsMetS_KD	1,7311	0,0817	3,3806	0,0477	0,2408	ns

CG-010_							
Bacteria_Bacillota_Clostridia_Peptococcales_Peptococcaceae_	MetS_SDvsMetS_KD	-0,5432	-1,8917	0,8053	0,4345	0,7048	ns
Peptococcus	MetS_SDvsMetS_KD	0,9798	-0,6294	2,5891	0,2405	0,5664	ns
Bacteria_Bacillota_Clostridia_Peptostreptococcales-Tissierellales_Anaerovoracaceae_	MetS_SDvsMetS_KD	0,8781	-1,3789	3,1351	0,4562	0,7240	ns
Anaerovorax	MetS_SDvsMetS_KD	-1,0785	-3,0795	0,9224	0,3013	0,6384	ns
Romboutsia	MetS_SDvsMetS_KD	0,0204	-1,1234	1,1642	0,9723	0,9888	ns
Bacteroides	MetS_SDvsMetS_KD	0,5064	-1,0817	2,0945	0,5353	0,7692	ns
Bacteria_Bacteroidota_Bacteroidia_Bacteroidales_Muribaculaceae_	MetS_SDvsMetS_KD	-0,7953	-1,8274	0,2368	0,1381	0,4098	ns
Muribaculum	MetS_SDvsMetS_KD	-0,3538	-2,3515	1,6440	0,7316	0,8613	ns
Prevotellaceae NK3B31 group	MetS_SDvsMetS_KD	-4,2976	-6,1629	-2,4323	0,0001	0,0024	**
Prevotellaceae UCG-001	MetS_SDvsMetS_KD	-2,3947	-3,8353	-0,9542	0,0022	0,0325	*
Alistipes	MetS_SDvsMetS_KD	-0,6239	-2,1140	0,8661	0,4164	0,6909	ns
Parabacteroides	MetS_SDvsMetS_KD	1,9451	0,5690	3,3212	0,0084	0,0764	ns
CAG-196	MetS_SDvsMetS_KD	1,9346	0,0107	3,8585	0,0587	0,2408	ns
Zag_111	MetS_SDvsMetS_KD	0,4363	-1,4650	2,3377	0,6556	0,8333	ns
Mucispirillum	MetS_SDvsMetS_KD	2,5107	0,6269	4,3945	0,0136	0,1102	ns
Bacteria_Pseudomonadota_Alphaproteobacteria_Rhodospirillales___	MetS_SDvsMetS_KD	3,1186	1,3655	4,8717	0,0014	0,0291	*
Parasutterella	MetS_SDvsMetS_KD	0,4466	-1,0785	1,9718	0,5690	0,7692	ns
Escherichia-Shigella	MetS_SDvsMetS_KD	2,5395	-0,8358	5,9147	0,1523	0,4098	ns
Bacteria_Thermodesulfobacteriota_Desulfovibrionia_Desulfovibrionales_Desulfovibrionaceae_	MetS_SDvsMetS_KD	2,7369	0,4795	4,9943	0,0226	0,1502	ns
Bilophila	MetS_SDvsMetS_KD	1,4207	-0,6519	3,4932	0,1880	0,4575	ns
Akkermansia	MetS_SDvsMetS_KD	1,6366	0,0345	3,2387	0,0514	0,2408	ns

Table S10.3 – Differentially abundant Genus identified using ANCOM-BC. Log fold changes (LFCs) and 95% confidence intervals are shown for each contrast. Multiple testing was controlled using the Benjamini–Hochberg false discovery rate (FDR); adjusted p-values (P_adj) are reported.

Taxon	Comparison	LFC	CI_low	CI_high	P_unadj	P_adj	Significance
Bifidobacterium	MetS_SDvsMetS_KS	-5,0994	-6,2642	-3,9345	0,0000	0,0000	***
Adlercreutzia	MetS_SDvsMetS_KS	-1,6754	-2,9491	-0,4016	0,0143	0,1424	ns
Bacteria_Bacillota_Bacilli_Erysipelotrichales___	MetS_SDvsMetS_KS	2,0102	0,0412	3,9791	0,0638	0,3328	ns
Bacteria_Bacillota_Bacilli_Erysipelotrichales_Erysipelotrichaceae_	MetS_SDvsMetS_KS	-3,2448	-4,6062	-1,8834	0,0000	0,0010	**
Allobaculum	MetS_SDvsMetS_KS	-0,6297	-2,3383	1,0790	0,4744	0,8658	ns
Dubosiella	MetS_SDvsMetS_KS	-1,6289	-3,0783	-0,1795	0,0352	0,2335	ns
Faecalibaculum	MetS_SDvsMetS_KS	-3,6738	-4,9517	-2,3959	0,0000	0,0001	***
Turicibacter	MetS_SDvsMetS_KS	-1,6696	-3,1986	-0,1406	0,0392	0,2384	ns
Bacteria_Bacillota_Bacilli_Izemoplasmatales___	MetS_SDvsMetS_KS	0,0455	-1,6190	1,7101	0,9577	0,9902	ns
HT002	MetS_SDvsMetS_KS	-0,2923	-1,3250	0,7404	0,5819	0,9300	ns
Lactobacillus	MetS_SDvsMetS_KS	-0,9990	-1,8721	-0,1260	0,0299	0,2335	ns
Bacteria_Bacillota_Bacilli_RF39___	MetS_SDvsMetS_KS	0,1773	-1,2382	1,5929	0,8073	0,9606	ns
Bacteria_Bacillota_Clostridia____	MetS_SDvsMetS_KS	0,7171	-0,9087	2,3430	0,3955	0,7674	ns
Christensenellaceae R-7 group	MetS_SDvsMetS_KS	0,8436	-0,3738	2,0610	0,1822	0,5542	ns
Bacteria_Bacillota_Clostridia_Clostridia UCG-014___	MetS_SDvsMetS_KS	0,0080	-1,1155	1,1315	0,9889	0,9902	ns
Bacteria_Bacillota_Clostridia_Clostridia vadinBB60 group___	MetS_SDvsMetS_KS	0,8664	-0,6826	2,4153	0,2801	0,7301	ns
Clostridium	MetS_SDvsMetS_KS	-2,1759	-3,6883	-0,6634	0,0071	0,0867	ns
Bacteria_Bacillota_Clostridia_Lachnospirales_Lachnospiraceae_	MetS_SDvsMetS_KS	-0,4814	-1,3700	0,4072	0,2943	0,7407	ns
[Eubacterium] xylanophilum group	MetS_SDvsMetS_KS	-0,3225	-2,0627	1,4177	0,7199	0,9606	ns
A2	MetS_SDvsMetS_KS	-1,2359	-2,7135	0,2417	0,1098	0,4716	ns
Acetatifactor	MetS_SDvsMetS_KS	0,1422	-1,7205	2,0050	0,8827	0,9649	ns
ASF356	MetS_SDvsMetS_KS	0,4295	-1,3347	2,1937	0,6379	0,9300	ns
Blautia	MetS_SDvsMetS_KS	-0,5635	-1,8325	0,7055	0,3896	0,7674	ns

Butyribacter	MetS_SDvsMetS_KS	-0,8876	-2,6402	0,8650	0,3304	0,7633	ns
Frisingicoccus	MetS_SDvsMetS_KS	1,3643	-0,3941	3,1227	0,1420	0,5182	ns
GCA-900066575	MetS_SDvsMetS_KS	0,4163	-1,2776	2,1101	0,6346	0,9300	ns
Lachnoclostridium	MetS_SDvsMetS_KS	0,1451	-1,2062	1,4964	0,8345	0,9606	ns
Lachnospiraceae AC2044 group	MetS_SDvsMetS_KS	1,2400	-0,4781	2,9582	0,1691	0,5366	ns
Lachnospiraceae NK4A136 group	MetS_SDvsMetS_KS	-0,1431	-1,1163	0,8301	0,7745	0,9606	ns
Mediterraneibacter	MetS_SDvsMetS_KS	0,5782	-1,2905	2,4468	0,5527	0,9270	ns
Roseburia	MetS_SDvsMetS_KS	-1,3574	-2,6952	-0,0196	0,0538	0,3020	ns
Monoglobus	MetS_SDvsMetS_KS	0,2423	-1,2885	1,7732	0,7584	0,9606	ns
Bacteria_Bacillota_Clostridia_Oscillospirales_[Eubacterium] coprostanoligenes group_	MetS_SDvsMetS_KS	0,6473	-0,6190	1,9136	0,3221	0,7633	ns
UCG-009	MetS_SDvsMetS_KS	0,1417	-1,2421	1,5254	0,8422	0,9606	ns
Bacteria_Bacillota_Clostridia_Oscillospirales_Oscillospiraceae_	MetS_SDvsMetS_KS	0,6985	-0,4150	1,8121	0,2262	0,6606	ns
Colidextribacter	MetS_SDvsMetS_KS	0,2808	-0,7964	1,3581	0,6122	0,9300	ns
Intestinimonas	MetS_SDvsMetS_KS	0,8059	-0,2214	1,8332	0,1318	0,5065	ns
NK4A214 group	MetS_SDvsMetS_KS	-0,5500	-1,7854	0,6854	0,3882	0,7674	ns
Oscillibacter	MetS_SDvsMetS_KS	-0,4135	-1,4379	0,6109	0,4334	0,8113	ns
UCG-003	MetS_SDvsMetS_KS	0,1618	-0,9784	1,3020	0,7823	0,9606	ns
UCG-005	MetS_SDvsMetS_KS	0,8843	-0,3406	2,1093	0,1641	0,5366	ns
Bacteria_Bacillota_Clostridia_Oscillospirales_Ruminococcaceae_	MetS_SDvsMetS_KS	0,4203	-0,8469	1,6876	0,5195	0,9250	ns
[Eubacterium] siraeum group	MetS_SDvsMetS_KS	-1,1477	-2,7144	0,4190	0,1608	0,5366	ns
Acutalibacter	MetS_SDvsMetS_KS	0,6530	-0,6847	1,9908	0,3451	0,7633	ns
Anaerofilum	MetS_SDvsMetS_KS	-0,6651	-2,7714	1,4412	0,5587	0,9270	ns
Anaerotruncus	MetS_SDvsMetS_KS	-0,2610	-1,7282	1,2062	0,7297	0,9606	ns
Fournierella	MetS_SDvsMetS_KS	0,4568	-1,6281	2,5418	0,6805	0,9553	ns
Paludicola	MetS_SDvsMetS_KS	0,0873	-1,8964	2,0711	0,9328	0,9869	ns
Pygmaiobacter	MetS_SDvsMetS_KS	0,1655	-1,6468	1,9777	0,8599	0,9649	ns
Ruminococcus	MetS_SDvsMetS_KS	-0,4064	-1,3251	0,5123	0,3908	0,7674	ns
Ruthenibacterium	MetS_SDvsMetS_KS	0,3771	-1,5536	2,3079	0,7076	0,9606	ns
Bacteria_Bacillota_Clostridia_Oscillospirales_U	MetS_SDvsMetS_KS	0,7289	-0,7355	2,1932	0,3364	0,7633	ns

CG-010_							
Bacteria_Bacillota_Clostridia_Peptococcales_Peptococcaceae_	MetS_SDvsMetS_KS	0,0813	-1,0192	1,1819	0,8856	0,9649	ns
Peptococcus	MetS_SDvsMetS_KS	0,5491	-0,7130	1,8111	0,3994	0,7674	ns
Bacteria_Bacillota_Clostridia_Peptostreptococcales-Tissierellales_Anaerovoracaceae_	MetS_SDvsMetS_KS	0,4619	-1,3827	2,3065	0,6298	0,9300	ns
Anaerovorax	MetS_SDvsMetS_KS	-0,2429	-1,9053	1,4195	0,7770	0,9606	ns
Romboutsia	MetS_SDvsMetS_KS	-1,3855	-2,2791	-0,4919	0,0039	0,0604	ns
Bacteroides	MetS_SDvsMetS_KS	0,7692	-0,1303	1,6688	0,1010	0,4716	ns
Bacteria_Bacteroidota_Bacteroidia_Bacteroidales_Muribaculaceae_	MetS_SDvsMetS_KS	-0,1849	-0,9775	0,6076	0,6497	0,9300	ns
Muribaculum	MetS_SDvsMetS_KS	-0,2011	-1,8517	1,4495	0,8133	0,9606	ns
Prevotellaceae NK3B31 group	MetS_SDvsMetS_KS	0,4400	-0,9311	1,8111	0,5333	0,9269	ns
Prevotellaceae UCG-001	MetS_SDvsMetS_KS	0,0193	-1,2705	1,3090	0,9768	0,9902	ns
Alistipes	MetS_SDvsMetS_KS	0,1116	-0,8932	1,1165	0,8287	0,9606	ns
Parabacteroides	MetS_SDvsMetS_KS	1,1180	0,1293	2,1067	0,0323	0,2335	ns
CAG-196	MetS_SDvsMetS_KS	-0,0112	-1,7806	1,7583	0,9902	0,9902	ns
Zag_111	MetS_SDvsMetS_KS	-0,8934	-2,3974	0,6107	0,2522	0,7076	ns
Mucispirillum	MetS_SDvsMetS_KS	-0,0659	-1,4876	1,3558	0,9282	0,9869	ns
Bacteria_Pseudomonadota_Alphaproteobacteria_Rhodospirillales___	MetS_SDvsMetS_KS	0,4611	-1,2054	2,1276	0,5912	0,9300	ns
Parasutterella	MetS_SDvsMetS_KS	-0,5888	-1,6035	0,4259	0,2617	0,7076	ns
Escherichia-Shigella	MetS_SDvsMetS_KS	1,3305	-0,3452	3,0061	0,1317	0,5065	ns
Bacteria_Thermodesulfobacteriota_Desulfovibrionia_Desulfovibrionales_Desulfovibrionaceae_	MetS_SDvsMetS_KS	1,0891	-0,2029	2,3811	0,1067	0,4716	ns
Bilophila	MetS_SDvsMetS_KS	2,4247	0,8792	3,9702	0,0041	0,0604	ns
Akkermansia	MetS_SDvsMetS_KS	1,6407	0,3624	2,9190	0,0156	0,1424	ns
